# Machine-learning-revealed statistics of the particle-carbon/binder detachment in lithium-ion battery cathodes

**DOI:** 10.1038/s41467-020-16233-5

**Published:** 2020-05-08

**Authors:** Zhisen Jiang, Jizhou Li, Yang Yang, Linqin Mu, Chenxi Wei, Xiqian Yu, Piero Pianetta, Kejie Zhao, Peter Cloetens, Feng Lin, Yijin Liu

**Affiliations:** 1 Stanford Synchrotron Radiation Lightsource, SLAC National Accelerator Laboratory, Menlo Park, CA 94025 USA; 2 Howard Hughes Medical Institute, Stanford University, Stanford, CA 94305 USA; 3 European Synchrotron Radiation Facility, Grenoble, 38000 France; 4 National Synchrotron Light Source II, Brookhaven National Laboratory, Upton, New York 11973 USA; 5 Department of Chemistry, Virginia Tech, Blacksburg, VA 24061 USA; 6 Beijing Advanced Innovation Center for Materials Genome Engineering, Institute of Physics, Chinese Academy of Sciences, Beijing, 100190 China; 7 School of Mechanical Engineering, Purdue University, West Lafayette, IN 47906 USA

**Keywords:** Batteries, Composites, Imaging techniques

## Abstract

The microstructure of a composite electrode determines how individual battery particles are charged and discharged in a lithium-ion battery. It is a frontier challenge to experimentally visualize and, subsequently, to understand the electrochemical consequences of battery particles’ evolving (de)attachment with the conductive matrix. Herein, we tackle this issue with a unique combination of multiscale experimental approaches, machine-learning-assisted statistical analysis, and experiment-informed mathematical modeling. Our results suggest that the degree of particle detachment is positively correlated with the charging rate and that smaller particles exhibit a higher degree of uncertainty in their detachment from the carbon/binder matrix. We further explore the feasibility and limitation of utilizing the reconstructed electron density as a proxy for the state-of-charge. Our findings highlight the importance of precisely quantifying the evolving nature of the battery electrode’s microstructure with statistical confidence, which is a key to maximize the utility of active particles towards higher battery capacity.

## Abstract (web summary)

Developing understanding of degradation phenomena in nickel rich cathodes is under intense investigation. Here the authors use learning-assisted statistical analysis and experiment-informed mathematical modelling to resolve the microstructure of a Ni-rich NMC composite cathode.

## Introduction

Lithium-ion batteries are regarded as a major breakthrough in the novel energy storage technology and have led to profound impacts on modern society. The energy storage and release in a lithium-ion battery involves lithium and electron transport between two electrodes, through the networks of electron-conducting carbon and ion-conducting electrolyte^1^. Such electron and lithium-ion (Li-ion) transport are driven by the externally applied electrical potential during charging and by the thermodynamically downhill reactions in the battery during discharging. An ideal composite electrode would offer a mechanically stable framework that allows for optimal electron and lithium-ion-conducting pathways, which entails the delicate control of the electrode microstructure through systematic electrode-scale studies. However, in contrast to the tremendous efforts in researching the active materials, fundamental experimental studies at the electrode level are relatively scarce, largely due to the lack of reliable experimental measurements of the particle behavior with spatial resolution and statistical relevance in complex, many-particle electrodes.

An in-depth understanding of the role that the electrode microstructure plays in modulating the battery performance requires thorough experimental input from advanced characterization methods^2^. Many of the electrode degradation mechanisms are directly associated with the spatial arrangement of different components in the electrode, including carbon matrix, void, binder, and active particles. Thus, the multiscale visualization of the composite electrode becomes crucial and is preferably done with sufficient spatial resolution and compositional sensitivity to resolve different components. Microstructural characterization of the composite electrode with desired representativeness, precision, reliability, and efficiency, however, is nontrivial. X-ray tomography^3,4^ has been widely adopted to conduct three-dimension (3D) tomographic imaging of the composite electrode in a number of different experimental modalities. For example, X-ray micro- and nano-tomography has been utilized to follow the dynamic evolution of the electrode materials under operating conditions at the electrode level^5–9^ and at the particle scale^10–13^. Coupled with the energy tunability of synchrotron X-rays, 2D/3D compositional^14,15^ and state-of-charge (SoC)^16–24^ heterogeneity can also be mapped out, providing valuable information about the local chemistry in lithium-ion batteries^25^. The conventional contrast mechanism based on the sample induced attenuation of the X-ray, however, clearly suffers from the limited capability of resolving the carbon/binder domain (CBD) in the electrode due to the weak-absorbing nature of the low Z elements (e.g., C and F). Therefore, the evaluation of the CBD’s role in the composite electrode has been performed either using modeling approaches^26^ or with other imaging techniques, e.g., focused ion beam and scanning electron microscopy^27,28^. However, the understanding of the particles’ electrochemical response to their respective local microstructural arrangement largely remains at a descriptive and speculative level. Experimental reconstruction of the microstructure of composite electrodes with nanoscale compositional and chemical sensitivity poses a frontier challenge in this field. We point out here that X-ray diffraction tomography^29^ and pair distribution function tomography, in which the spatially resolved X-ray diffraction signal is recorded as the sample is raster scanned and rotated, have also been utilized to look into the structural heterogeneity in battery materials under operating conditions^30–32^. While these techniques are sensitive to the atomic arrangements of the material’s lattice structure, the effective spatial resolution is often determined by the X-ray focal spot used to raster scan the sample and is typically only at the micron-level due to practical experimental constraints, such as inferior data collection speed.

Herein, we tackle this challenge by conducting high-resolution hard X-ray nano-tomography based on the phase contrast modality. We demonstrate the visualization of active particles, CBD, and pore structures in a Ni-rich LiNi_0.8_Mn_0.1_Co_0.1_O_2_ (NMC) composite cathode at the charged state. Through 3D reconstructing and visualizing the physical contact between the active NMC particles and the conductive CBD matrix, we model the spatial heterogeneity of the local electrical resistance over the surface of individual particles. Our modeling result suggests that the detachment of the active NMC particles from the CBD influences the homogeneity of the electrical resistance over the particle surface, which could substantially impact the particle’s participation in the cell level chemistry. To ensure the statistical representativeness, we develop a machine learning model that conducts the identification and quantification of over 650 NMC particles automatically. The machine-learning-assisted statistical analysis reveals that the degree of particle detachment from the CBD is dependent on the charging protocol and the particle size. Our large-scale, many-particle approach has eliminated the potentially biased characterization results reported using conventional image techniques, which usually accounts for a limited number of particles. We also demonstrate that the quantitative phase retrieval is capable of reconstructing the spatial distribution of the electron density over the imaged sample volume. Under reasonable approximations, we could link the electron density to the SoC, which is confirmed by the Ni valence state mapping using the X-ray spectro-microscopy technique in a correlative imaging manner. Although the trend of the particles’ averaged electron density as a function of their respective detachment is not obvious in our statistical analysis, possibly due to the relaxation of the electrode after it was disassembled from the cell, we highlight here that the sub-particle level charge heterogeneity could persist. Such a phenomenon is attributed to the sub-particle level structural and chemical defects, which could lead to thermodynamically stable charge heterogeneity and play a significant role in the active particle degradation.

## Results

### Visualizing the NMC cathode using phase contrast tomography

The composite cathode is made of active Ni-rich NMC particles that are embedded in the porous CBD matrix. More details about the electrode synthesis can be found in the Methods section. In addition to the mechanical support, the CBD matrix also provides interconnected conductive networks for electrons and the Li-ions, respectively, through the carbon phase and the void space that is filled with the liquid electrolyte. In order to reveal the 3D microstructure and composition with high spatial resolution down to the nanoscale, we employed the hard X-ray phase contrast nano-tomography technique at the beamline ID16A-NI at the European Synchrotron Radiation Facility (ESRF). Using an X-ray focal spot of around 20 nm as the secondary source^33^, this method allows for 3D non-invasive virtual imaging with quantitative phase contrast instead of attenuation contrast. The recovered phase contrast maps^34^ are proportional to the real part of the complex refractive index, which is determined by the local electron density of the materials. Due to this, the phase contrast modality, when compared to attenuation-based contrast, can offer substantially improved sensitivity when using high energy X-rays for measurement, in particular for the light (low Z) materials. Hence both the light (low Z) materials and the heavy metal oxides (NMC particles) can be visualized with hard X-ray phase contrast imaging with adequate quantitative sensitivity.

**Fig. 1:**
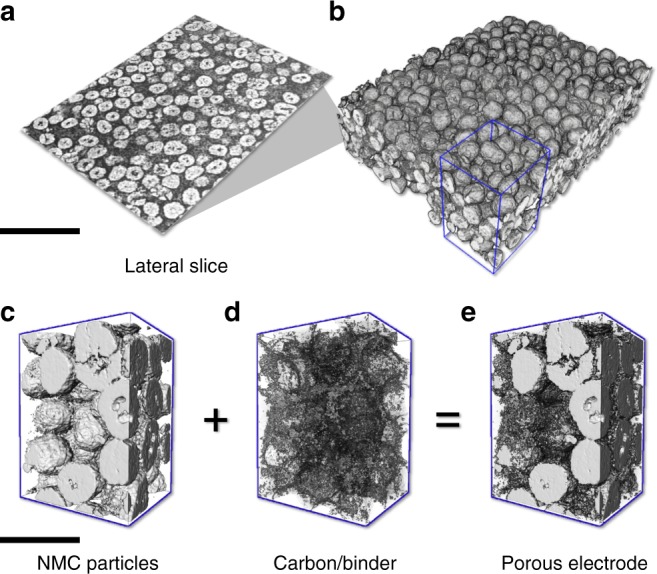
3D microstructure of the composite battery cathode. **a**, **b** An overview of a relatively large area on the electrode, which covers hundreds of NMC particles, with the central lateral virtual slice displayed in (**a**). **c**–**e** A smaller region of interest (the region highlighted by the blue box in (**b**)) with the active NMC particles (**c**) and the inactive carbon/binder domains (**d**) rendered separately and jointly (**e**). The scale bars in (**a**) and (**c**) are 60 and 20 μm, respectively.

As highlighted in Fig. 1a, b (the 3D volume rendering and a representative virtual slice), our data clearly resolves the sub-particle level microstructure of many particles at once, offering a significant amount of microstructural information for a systematic statistical analysis. With the quantitative phase contrast modality^34^, we are able to distinguish and to segment the active particles, the CBD, and the pore network with excellent accuracy (see Supplementary Fig. 1 for the segmentation process). We show in Fig. 1c–e a magnified view of an arbitrarily selected sub-region, which further demonstrates the complexity in the spatial arrangement of the different components in the studied composite electrode. It is worth noting that the spatial resolution of our data is not sufficient to resolve the fine pore structure within the CBD (see Supplementary Fig. 1). The present work focuses on the relatively large features caused by the NMC particle detachment from the CBD network, which can be visualized and quantified with good fidelity. We also point out that a depth-dependent damage profile has been observed and reported (see Supplementary Fig. 2 for the comparison between the top and the bottom of the electrode^35,36^). To rule out this effect and to simplify the interpretation, we will utilize a thin electrode with a monolayer of active NMC particles in the following studies.

### Modeling local electrical resistance over the particle surface

**Fig. 2:**
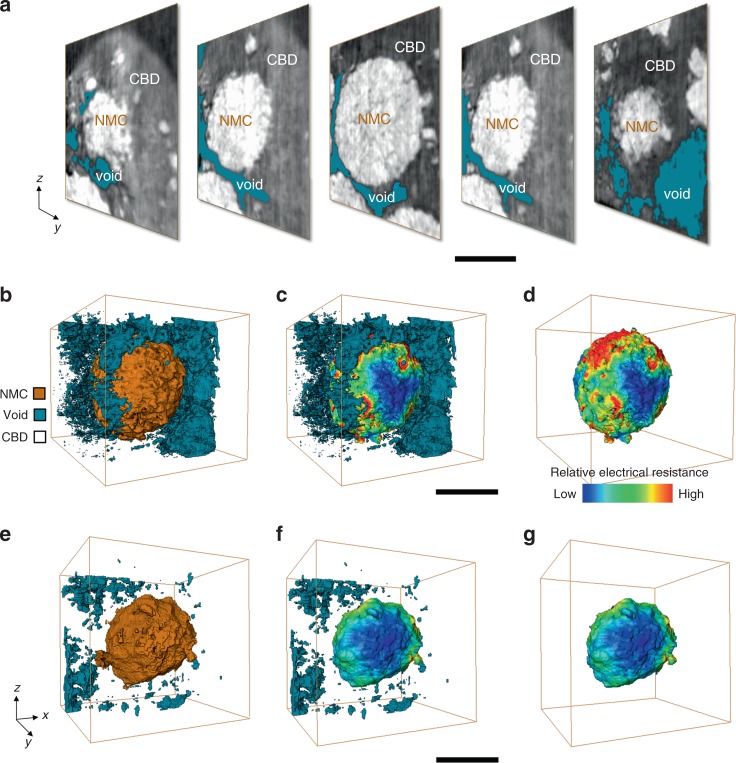
Visualization and modeling of the particle detachment. **a** Selected *y*–*z* slices through an NMC particle. **b**, **e** The 3D rendering of the segmentation results over two regions of interest, with the CBD set to be transparent for a better visualization of the NMC particle (orange) and the voids (gray–blue). **c**, **f** The renderings of the calculated distributions of relative local electrical resistance over the surface of the particles in (**b**) and (**e**), respectively. Our modeling result suggests a strong correlation between the degree of CBD attachment and the level of calculated electrical resistance heterogeneity. **d**, **g** The same particles of the (**c**) and (**f**) presented without the void phase. The scale bar in (**a**), (**c**), and (**f**) are 10, 10, and 5 μm, respectively.

As discussed above, the phase contrast methodology allows for visualizing the degree of active NMC particle’s detachment from the conductive CBD. The virtual slices through different depths of a selected NMC particle is shown in Fig. 2a with the gap between the particle and the CBD highlighted in gray–blue color and labeled as “void”. These gaps are infiltrated by the liquid electrolyte, which conducts the Li-ions but not the electrons, causing the heterogeneity in the local electrical and ionic resistance over the particle surface. As a consequence, during the charging process, the electrons need to detour around the (electrolyte filled) voids and reach to the nearest electrical contact point before they could enter the conductive CBD network. The electrical resistance of the active material is often considerably higher than that of the CBD, and is actually a function of the SoC^37,38^, further complicating the scenario.

For better understanding such effect, we developed a numerical model to calculate the spatial distribution of the relative electrical resistance over the surface of the NMC particles based on the phase contrast tomography data. We use the detachment-induced detouring distance for the electron diffusion as a proxy for the local electrical resistance (see schematic illustration in Supplementary Fig. 3). Two particles with different degrees of detachment are shown in Fig. 2b (same as the one in Fig. 2a, showing considerable particle detachment from the CBD) and Fig. 2e (well intact with the CBD) as the examples. The calculated distribution of the relative local electrical resistance over these two selected particles are presented in Fig. 2c, f, respectively. Our modeling results suggest that the particle (Fig. 2b–d) that is partially detached from the CBD has developed significant local electrical resistance (red regions), while the particle (Fig. 2e–g) that is mostly intact seems to be affected only slightly. We anticipate that the ionic conductivity over the particle surface is inversely correlated with the electrical conductivity calculated here. This is because the gap between the particle and the CBD is infiltrated with ion-conducting electrolyte. The partial detachment of NMC particles from the CBD network leads to rearrangement of the local current density distribution, which could significantly impact the health of the corresponding particle and, subsequently, that of the cell. The results of our study are highly relevant to the commonly used calendering process for electrode production^39^.

### Machine-learning-assisted statistical analysis

**Fig. 3:**
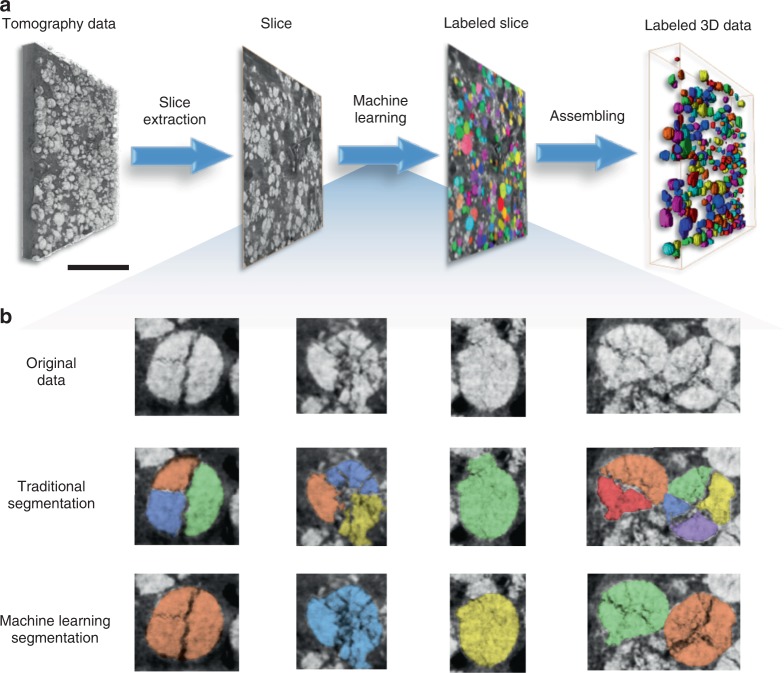
Machine learning-based segmentation and labeling. Over 650 unique particles of different size, shape, position, and degree of cracking were successfully identified and isolated from the imaging data in an automatic manner. **a** Workflow of the machine learning-based segmentation. **b** Comparison of conventional segmentation results and the machine-learning-assisted segmentation results for a few representative particles. Different colors denote different particle labels. The scale bar in (**a**) is 50 μm.

While the above discussed particle-level modeling results are very valuable, the statistical representativeness is another vital factor for a thorough investigation considering the complexity of the composite electrode. Our phase contrast tomographic result covers more than 650 active particles (over multiple fields of view). The identification and segmentation of every individual particle in the reconstructed 3D volume is, however, tedious and labor-intensive in the conventional manual approach. The automation of this process is nontrivial, in particular for the severely damaged particles. The identification of multiple fragments that broke away from the same particle requires some level of “intelligence” and, therefore, often requires human involvement in the process. To tackle this problem, we developed a machine learning approach that automatically accomplishes the task with superior accuracy and efficiency. The high-level workflow is outlined in Fig. 3a and the underlying machine learning model is illustrated in Supplementary Fig. 4. More descriptions of our machine learning approach can be found in the Methods section. To highlight the robustness of the developed method, we show in Fig. 3b the comparison of the segmentation results on a few representative particles using conventional approach (watershed and separation algorithms) and our new machine learning method. Although the exceptional image contrast facilitates the identification of the NMC phase with good accuracy, the conventional segmentation method clearly failed to separate different particles and, in contrast, our machine learning approach demonstrates significantly improved performance.

**Fig. 4:**
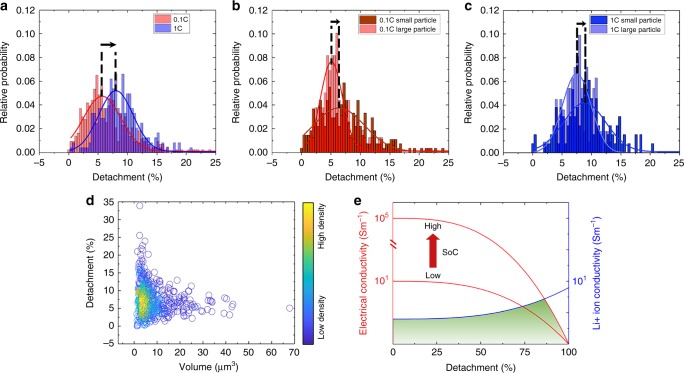
Statistical analysis of the particle detachment. Statistical comparisons of the degree of particle detachment from the CBD matrix are carried out as a function of cycling rate (**a**) and particle size (**b**, **c**), respectively. The degree of particle detachment is also plotted against the respective particle volume in (**d**) for all the 650 NMC particles studied in this work. **e** Schematic of the changes of the electrical (red) and ionic (blue) conductivity as a function of the particle detachment. The red curve moves up and down depending on the SoC. The particle’s actual contribution falls in the green shaded area.

Build upon the machine-learning-assisted segmentation result, we quantified the characteristics of every single NMC particles including their degree of detachment (defined by the ratio of the detached particle surface, 0% means fully intact and 100% would be completely detached), volume, and relative intensity. We compared the data from electrodes that were subjected to different cycling protocols (1 and 0.1 C, see Supplementary Fig. 5). Over 650 NMC particles in total were analyzed and the probability distribution of the degree of detachment is compared between the fast (1 C, blue) and slow (0.1 C, red) cycled particles (see Fig. 4a). We clearly observed a shift of the peak in the probability distribution, suggesting that the particles undergoing a faster charging rate experienced more severe detachment. Such an effect could be part of the reason for the rapid capacity decay under fast charging conditions. To elucidate the particle size dependence of the detachment effect, we divided the particles into two groups (big and small) based on their respective volumes. The big particles are ≥11 μm in diameter, and the small particles are <11 μm in diameter (the threshold is based on the statistical analysis of the particle size distribution). The relative probability distribution of the particle detachment in the big and the small groups are compared for the fast (1 C, Fig. 4c) and slow (0.1 C, Fig. 4b) cycled electrodes, respectively. Interestingly, in both cases, the small particles demonstrate a more broadly scattered probability distribution with the peak position slightly shifted to the right (as shown by the black arrow). We further plotted the degree of detachment for all the particles versus their respective volume in Fig. 4d. This scattered plot further demonstrates the uncertainty in the particle detachment as a function of the particle volume. This result suggests that the small particles have a larger degree of uncertainty in terms of the physical detachment from CBD, which is a piece of very valuable information that could inform the engineering effort to optimize the electrode formation for fast charging applications.

### NMC particle’s response to the detachment from the CBD

We show in Fig. 4e a conceptual illustration of the change of the electrical and ionic conductivity as a function of the degree of the particle detachment from the CBD. As discussed through our modeling results, such detachment clearly rearranges the local electrical conductivity. On the other hand, it favors the local ionic conductivity as it facilitates better contact between the liquid electrolyte and the NMC particle. These two competing factors collectively govern the particle’s behavior. A particle’s actual contribution to the cell level chemistry is dominated by whichever is worse. As a consequence, the performance of the particle could slightly improve in the early stage of the particle detachment due to the improved ionic conductivity. In more severely detached circumstances, the decreased electrical conductivity takes over and results in an overall negative impact. We note that the electrical conductivity of the NMC material actually changes significantly as a function of SoC^40^. Therefore, the red curve in Fig. 4e moves up and down upon cycling and the green shaded area, which illustrates the particle’s actual contribution, would vary accordingly. Nevertheless, the mild particle detachment could favor the balanced electrical and ionic conductivity of the particle. We point out that, beyond the diffusion kinetics, the particle detachment could have a negative impact from the mechanical perspective. Although it is beyond the scope of the present work, we believe that it is an area worth further in-depth investigation.

### Correlation of the local electron density and the local SoC

**Fig. 5:**
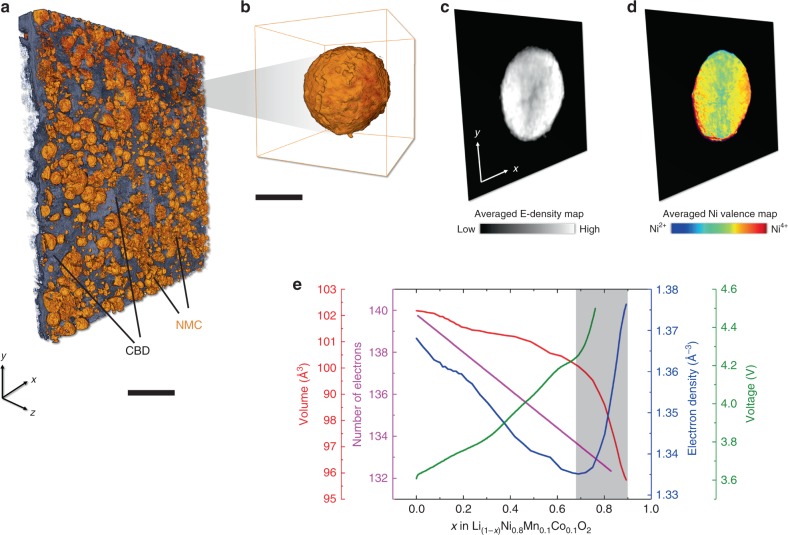
Electron density as a proxy for the SoC. **a** The 3D rendering of an electrode with a monolayer of NMC 811 particles. **b**–**d** illustrates our correlative approach for imaging the same NMC particle. **c** The phase contrast image collected at ID16A, ESRF, Grenoble, France. **d** The spectroscopic imaging data of the same particle collected at 6-2C, SSRL, Menlo Park, CA, USA. **e** shows the changes of lattice unit cell volume, number of electrons per lattice unit cell, voltage (1 C), and electron density as a function of the SoC. The scale bars in (**a**) and (**b**) are 25 and 5 μm, respectively.

Another advantage of the employed X-ray phase contrast methodology is the capability of quantitatively retrieving the electron density distribution^34,41^ over the studied volume. The NMC’s electron density is a fundamental physical property that changes upon charging and discharging. The total number of electrons within a unit cell of the NMC lattice decreases upon charging because electrons and lithium ions are both extracted from the cathode. The average number of electrons per lattice unit cell can be quantified by integrating all the electrons in the NMC unit lattice cell at different SoCs (note that the formula for an NMC 811 unit cell is Li_3−3*×*_Ni_2.4_Mn_0.3_Co_0.3_O_6_), which is plotted in Fig. 5e as a function of *x*, i.e., the SoC. On the other hand, as the lithium ions diffuse from the NMC lattice into the electrolyte, the NMC lattice shrinks in an anisotropic manner. The change of the NMC’s lattice parameters (*a*, *b*, and *c*) as a function of the SoC has been reported through *operando* monitoring of the Bragg diffraction peaks^42^. The volume of the NMC unit cell can, therefore, be calculated and plotted against the SoC (Fig. 5e). Subsequently, we show in Fig. 5e the calculated change of electron density in the NMC lattice as a function of the SoC. At low SoC (smaller *x* value), the electron density decreases upon charging (increasing *x*). At *x* = ~0.7, there is a turning point, beyond which the electron density increases rapidly upon charging. This is because the NMC lattice parameter changes rapidly at high SoC, which is part of the reason for the instability of the NMC cathode at a deeply delithiated state. It is worth noting that the relative changes in both the lattice unit cell volume (up to ~5.9%) and the total number of electrons in a unit cell (up to ~5.7%) are at a similar level and, thus, jointly affect the effective electron density. We also acknowledge that the electron density is not monotonically correlated with the SoC and, therefore, it cannot be used to quantify the SoC universally. However, at the deeply charged state (gray area in Fig. 5e; our electrodes were harvested after the cell was charged to 4.5 V (at *x* equals 0.75–0.8)), we can assume the one-to-one correlation because the coexistence of the deeply charged and discharged states (very high and very low *x* values) is highly unlikely unless there is a very severe deactivation effect that completely isolates the local domain from the rest of the electrode.

For better verification of the correlation between the electron density and the SoC, we carried out correlative phase contrast (ID16A, ESRF, Grenoble, France) and spectroscopic (6-2C, SSRL, Menlo Park, CA, USA) X-ray imaging (see schematic illustration in Supplementary Fig. 6) on the same NMC particle (Fig. 5b) that is arbitrarily selected from the electrode (Fig. 5a). The X-ray spectro-microscopy is a well-established tool for probing the local oxidation state of the element of interest^18,22^. In our case, we focused on the Ni K-edge because the redox reaction of the Ni cation is the major contributor to the charge compensation in the Ni-rich NMC cathode^43^ and, therefore, the Ni valence state can be used as a proxy for the local SoC of the NMC cathode^44–47^. As shown in Fig. 5c, d, the depth-averaged projection view of the particle after charging to 4.5 V shows reasonable similarity (the Pearson correlation coefficient is quantified to be ~0.54) between the electron density map (Fig. 5c) and the Ni oxidation state map (Fig. 5d). The detailed data reduction and normalization procedures for both techniques are illustrated in Supplementary Fig. 7. Our result suggests that the quantitatively retrieved electron density map from the X-ray phase contrast methodology can potentially be utilized to evaluate local SoC distribution under reasonable assumptions.

Finally, we quantify the electron density for all the machine-learning-identified NMC particles in our tomographic data. As shown in Supplementary Fig. 8, the particles’ electron densities are plotted against their respective degree of detachment from the CBD. The data points appear to be quite scattered and we do not observe a clear trend in this plot. This is possibly caused by the relaxation of the electrode after it is disassembled from the cell, which urges for a follow-up phase contrast imaging study of the battery electrode under *operando* conditions. Another possible explanation for the unclear trend is due to the non-monotonicity of the electron density versus the SoC, which adds some degree of ambiguity in particular near the turning point in Fig. 5e (*x* near 0.7). We anticipate that such quantification will be more reliable at the intermediately charged state due to the relatively large SoC window with monotonic electron density evolution (0 < *x* < 0.7).

## Discussion

The microstructure of composite electrodes plays a significant role in affecting the overall performance of the lithium-ion batteries. Direct measurement of the structural and chemical heterogeneity in the NMC cathode could offer valuable insights into the interplay among the local electrical resistance, the particle morphology (size, shape, and cracks), the arrangement of the ionic conductive network, and the local potential gradient, which collectively affects the particle’s response to the operation conditions and, subsequently, influences the cell chemistry. With the quantitative X-ray phase contrast nano-tomography technique, we first visualize the NMC active particle’s detachment from the CBD. A numerical model is developed to calculate the spatial heterogeneity of the electrical conductivity over the surface of the partially detached particles. To carry out this quantification with better statistics, we developed a machine learning model, which identified and segmented over 650 NMC particles automatically. Our statistical analysis shows that the fast-cycled particles exhibit more severe detachment from the CBD and the smaller particles exhibit a higher degree of uncertainty in their CBD detachment. We further explored the possibility of utilizing the reconstructed local electron density as a proxy for the local SoC. Our results confirmed the feasibility and also pointed out some limitations of this approach. Combining the cutting edge experimental, modeling, and machine learning capabilities, our work sheds new light on the fundamental mechanism behind the complicated relationship between the microstructure of the composite electrode and the performance. Our work highlights the importance of balanced diffusion kinetics for both charge carriers (Li-ions and electrons) for optimal battery performance. Such a criterion is particularly important for guiding the design of next-generation Li-ion batteries with fast charging capability.

There are few limitations with the current procedure. First of all, the disassembling process could cause damage to the electrode and, subsequently, affect the result of the statistical analysis. Second, the relaxation of the electrode may lead to charge redistribution in the electrode, making it difficult to evaluate the electrode scale chemical heterogeneity, which could be thermodynamically metastable. Finally, the cell-to-cell discrepancy is a common effect, which could add more complexity to the analysis and interpretation. All these limitations can be tackled by implementing an operando experimental strategy.

Looking forward, the presented development of automatic segmentation and local resistance modeling capability set the basis for a number of follow-up studies. For example, our segmentation approach could take the human out of the loop in analyzing a massive amount of data, and subsequently, could facilitate more sophisticated statistical analysis including the correlations of many different morphological characteristics and the chemomechanical breakdown of the particles. We could systematically evaluate the particle-size-dependence, the sphericity-dependence, the porosity-dependence, the particle-to-particle interaction, to name a few. The importance of this research direction is caused by the intrinsic complexity in the morphology–performance relationship. Such a complicated effect relies on a thorough analysis with statistical significance.

## Methods

### NMC composite electrode synthesis

The composite cathodes were prepared by spreading the slurry (N-methyl-2-pyrrolidone as the solvent) with active materials (90 wt%), acetylene carbon (5 wt%), and polyvinylidene difluoride (5 wt%) as the binder and casting them on carbon-coated aluminum foils. The electrodes were then dried overnight at 120 °C in a vacuum oven and transferred into an Ar-filled glove box for future use. Two cells were both cycled under C/10 for the first cycle and 1 C for the second cycle as an activation process. After that they were, respectively, subjected to 10 cycles under C/10 and 1 C, respectively. For the ex-situ measurements, the electrodes were then disassembled in an Ar-filled glove box. We show, in Supplementary Fig. 5, the voltage–capacity curves of the two cells used in this study, which suggests that the cell underwent gradual capacity fading. Nevertheless, at the end of the designed cycling sequence, we reach a state of voltage at 4.5 V and *x* at 0.75–0.8. For correlative X-ray phase contrast nano-tomography and X-ray spectro-microscopy, the sample was a small piece cut carefully from a complete cathode plate, then fixed on top of a Huber pin in order to mount in the rotation stage. The tomography scan was conducted near the center of the piece, away from the cut edges to avoid any sample prep induced artifacts. All the samples were protected in the inert gas environment during storage, transportation, handling, and measurements.

### Nano-resolution X-ray spectro-microscopy

We conducted X-ray spectro-microscopic scan of the deeply charged Li_0.5_Ni_0.8_Mn_0.1_Co_0.1_O_2_ particles using the transmission X-ray microscopy (TXM) at beamline 6-2C of Stanford Synchrotron Radiation Lightsource of the SLAC National Accelerator Laboratory. The typical exposure time for single images is 0.5 s. The nominal spatial resolution of this instrument is ~30 nm. More details of the synchrotron beamline configuration and the concept of X-ray spectro-microscopy and spectro-tomography can be found elsewhere^17^. In the 2D spectro-microscopic scan, the energy of the incident X-rays is scanned from 8200 to 8630 eV to cover the absorption K-edges of Ni with over 150 energy points. In the near edge region (8330–8355 eV), we chose the energy step at 1 eV to ensure sufficient energy resolution. The pre-edge and post-edge regions were scanned with larger energy steps of 10 eV to cover a relatively wide energy window for normalization of the spectra. The TXM data processing was performed using an in-house developed software package known as TXM-Wizard^18^. The segmentation and visualization of the 3D data were carried out using a commercial software package known as Avizo. We highlight here that a small piece of the NMC cathode electrode is mounted on a Huber pin that is compatible with and is transferred between the ID-16 of ESRF and 6-2C of SSRL for correlative imaging of the same particle.

### X-ray phase contrast nano-tomography

The X-ray phase contrast nano-tomography measurements were conducted at the ID16A-NI nano-imaging beamline at the European Synchrotron Radiation Facility (ESRF) in Grenoble, France. This beamline features a high energy hard X-ray nano-probe, delivering a focus down to ~20 nm with a brilliant photon flux (up to 10^12^ photons/s at Δ*E*/*E* ~ 1%). The nano-focus is achieved by two pairs of multilayer-coated Kirkpatrick–Baez (KB) optics, working at 17 and 33.6 keV respectively. In our measurements at 17 keV, the sample was placed downstream of the KB focus and magnified radiographs were recorded onto an X-ray detector using a FReLoN charged-coupled device with a 2048 × 2048 binned pixels array. After the magnification, the effective pixel size is equivalent to 50 nm. For every tomography scan, 1500 projections were acquired with 0.2 s exposure time. One complete phase contrast nano-tomography scan constitutes tomograms at four different focus-to-sample distances. These tomograms were subsequently used for phase retrieval to generate 2D phase maps^34^. The 2D phase maps retrieved from all angular projections were then used as input for a tomographic reconstruction based on the filtered back projection algorithm method (ESRF PyHST software package)^48^. The reconstructed 3D phase contrast volumes are proportional to the electron density of the sample.

### Machine-learning-based identification and segmentation

Manual annotations on a set of nano-tomographic slices of thick NMC composite electrodes (see Fig. 1a) were used to train a machine-learning model, which was then applied to the current dataset of monolayer NMC electrode. Each slice of the reconstructed volume is processed separately, and the obtained 2D masks are combined to reconstruct the 3D particles afterward. The instance-aware identification and segmentation of NMC particles in each slice are accomplished using a state-of-the-art mask regional convolutional neural network (Mask R-CNN)^49^. Intuitively, this model takes advantage of the inherent hierarchical and multi-scale characteristic of a convolutional neural network to derive useful features for object detection. Rather than training the network end-to-end from the start, the ResNet-101 feature pyramid network was used and the model was initialized by the weights obtained from the large-scale ImageNet dataset (a widely used on-line database for benchmarking of the object detection algorithms). Starting from the pre-training weights, we optimize the network by incorporating the information of the NMC particle shape as defined in the manually annotated data. Such an approach effectively adds additional constraint in the segmentation and reinforces the overall quasi-spherical shape of the particles. A schematic illustration of the machine learning model architecture is presented in Supplementary Fig. 4. Methodologically, the Mask R-CNN first generates regions of proposals (i.e., candidate bounding boxed for particles) after scanning the image from the convolutional feature maps; and it then predicts the bounding box and binary mask for each particle by searching on feature maps. After segmentation, each mask contains a particle’s outline bearing a unique identifier. Those identifiers are then used for linking slices at different depths of the volume to construct 3D particles for further analysis by the Hungarian maximum matching algorithm^50^.

The traditional watershed algorithm relies on the inner distance map as marking function and easily causes over-segmentation and/or under-segmentation when the boundaries of NMC particles are not clear or the signal-to-noise ratio of the image is low. More importantly, the particle’s external boundary (versus the surface of the pores and cracks) cannot be easily defined in the conventional approach that is simply based on the local pixel intensity. Therefore, the formation of cracks in the particles (low-intensity features) could significantly and falsely alter the inner distance map and such an effect cannot be addressed by improving the image quality. As a result, due to the mechanical disintegration of the NMC particles, the traditional algorithm often mistakenly splits one particle into several parts (see Fig. 3b) with different labels assigned.

Our results suggest that this approach shows significantly improved robustness against the formation of the inner-particle cracks, which would otherwise result in the identification of smaller irregularly shaped parts. For better illustration of this statement, we show in Supplementary Fig. 9a the comparison of the input image and the activation map, which is extracted from an intermediate layer of our network. We point out here that only the particles’ external boundaries are highlighted. The emphasis of the particles’ external boundaries with simultaneous suppression of the crack surface is exactly the desired functionality of the auto segmentation algorithm and it is not possible to achieve such a purpose purely based on the intensity values of the input image. For a more quantitative comparison of the results from the conventional watershed segmentation and the herein developed Mask R-CNN algorithm, we show in Supplementary Fig. 9b six different evaluation metrics. It is evident that our approach significantly outperforms the conventional method in all of these aspects. The detailed description of the training procedure and these evaluation metrics are included in Supplementary Note 1.

We would also point out that, with our development, the current network can be further optimized when a new dataset comes in. The re-optimization process of the algorithm requires only a very small amount of training data.

## Supplementary information

Supplementary Information

Peer Review File

## Data Availability

All data that support the findings of this study are available from the corresponding authors upon reasonable request. The nano-tomography data sets used for the machine learning development are made publicly available at GitHub repository: https://github.com/hijizhou/LIBNet.

## Code availability

The source code and detailed instructions are made publicly available at the GitHub repository: https://github.com/hijizhou/LIBNet.
