# Supplementary material for: Machine-learning-revealed statistics of the particle-carbon/binder detachment in lithium-ion battery cathodes
Source: Nat Commun. 2020 May 8;11:2310. doi: 10.1038/s41467-020-16233-5 (PMC7210251; doi:10.1038/s41467-020-16233-5)
Supplement: Supplementary file 1 — Supplementary Information [file 41467_2020_16233_MOESM1_ESM.pdf]

Machine-learning-revealed statistics of the particle-carbon/binder detachment in lithium-ion battery cathodes

Jiang et al.

Supplementary Figures

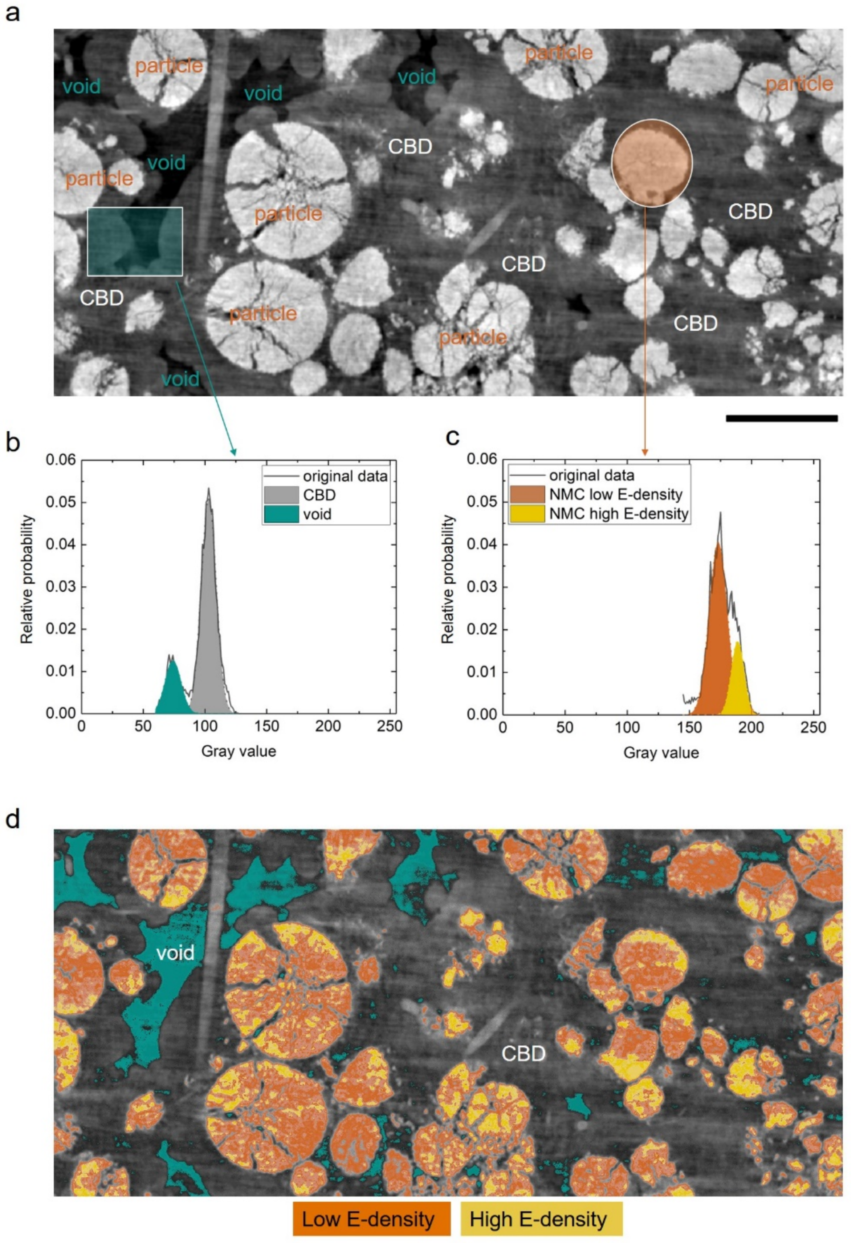

Supplementary Figure 1. Segmentation of the phase contrast x-ray nano-tomographic data. (a) shows a typical slice through the center of the electrode. (b) and (c) show the histograms over different regions on the image. The excellent image contrast offered by the quantitative phase contrast methodology enables the segmentation of the void, the CBD, and regions with low and high electron densities in the active particles ((d)) with good fidelity. The scale bar in (a) is 15 µm.

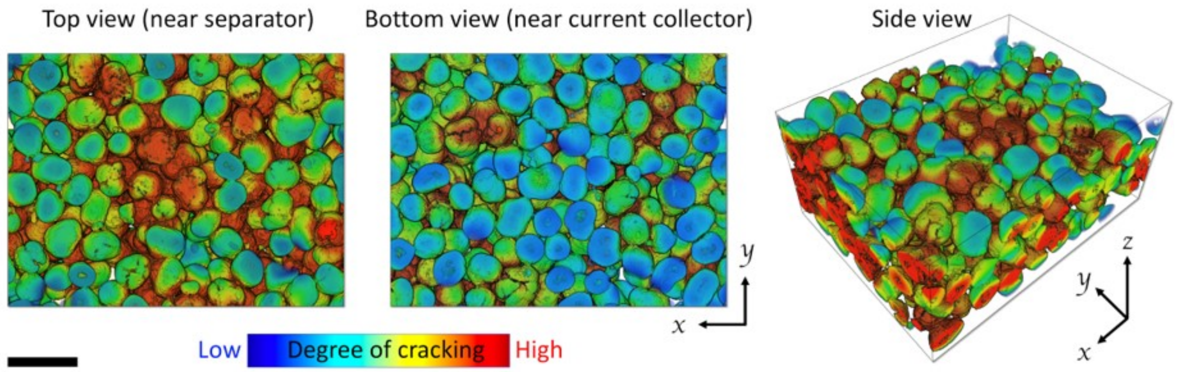

Supplementary Figure 2. A depth dependent particle fracturing profile in Ni-rich NMC composite electrode revealed by X-ray nano-tomography data. The image is color coded to the local degree of morphological damage, which is quantified by evaluating the local concentration of crack surface. The scale bar is 20 μm.

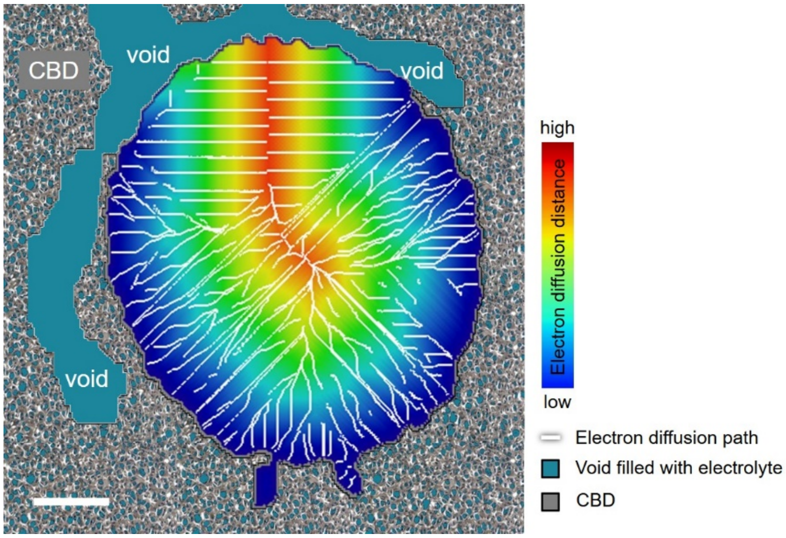

Supplementary Figure 3. Schematic drawing of the herein developed numerical model for calculating the diffusion pathways for electrons within the NMC particle. The particle is embedded in the porous CBD matrix and is partially detached from the CBD network. The void space is infiltrated by the liquid electrolyte, which favors the local lithium diffusion kinetics, but detours the electrons before they could reach the CBD network. The particle is color coded to the electron's diffusion length and the white lines over the particle illustrate the local gradient of the diffusion length distribution which indicates the geometrical optimal diffusion path of the electron over the particle. We point out here that this schematic drawing shows a 2D scenario for easier presentation. Our actual calculation was conducted in the 3D space. Note that the model considers the NMC particle as a continuous solid and the electronic conductivity is uniform and isotropic in the particle. The scale bar is 3 μm.

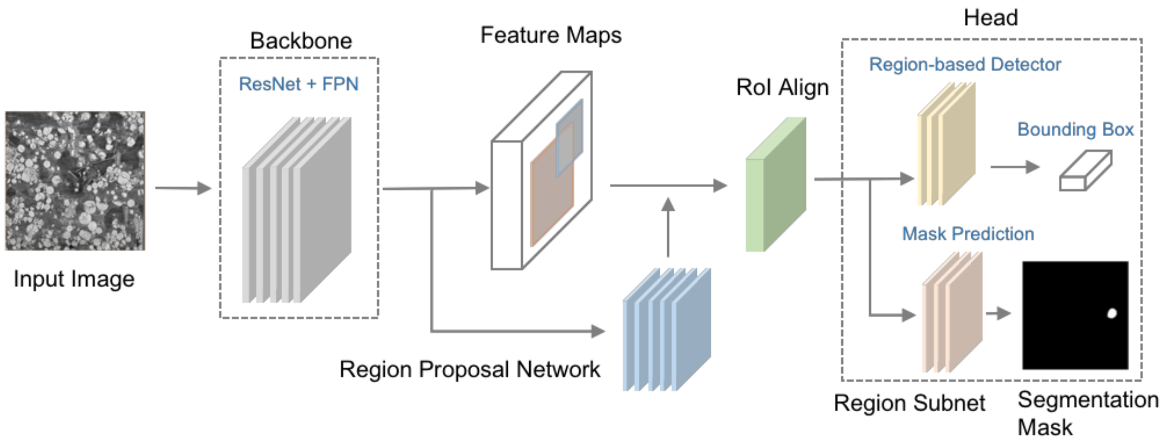

Supplementary Figure 4. Schematic illustration of the herein developed machine learning model based on the Mask R-CNN for particle identification and segmentation. The model facilitates the detection of over 650 active particles in our phase contrast tomographic result, which set the basis for our statistical analysis. For the input slice, the residual neural network (ResNet) and feature pyramid network (FPN) are utilized as the backbone for feature extraction at different scales. After alignment of region-of-interest (RoI) with the extracted features, the head sub-network predicts bounding boxes for particles and then segments the particle inside the predicted boxes as a binary mask.

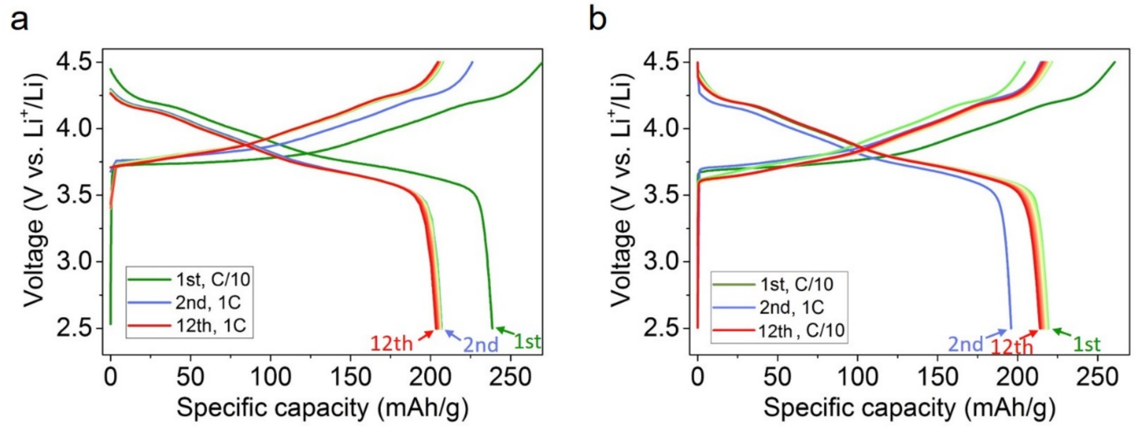

Supplementary Figure 5. Electrochemical data of the cells studied herein. The two cells were cycled under C/10 for the first cycle and 1C for the second cycle for an activation process. After that they were subjected to 10 cycles under 1C (a) and C/10 (b), respectively. We point out here that these mono layer electrodes have very low mass loading of 1 mg/cm^2. The discrepancy in the initial capacity is likely caused by the experimental errors due to the low mass loading.

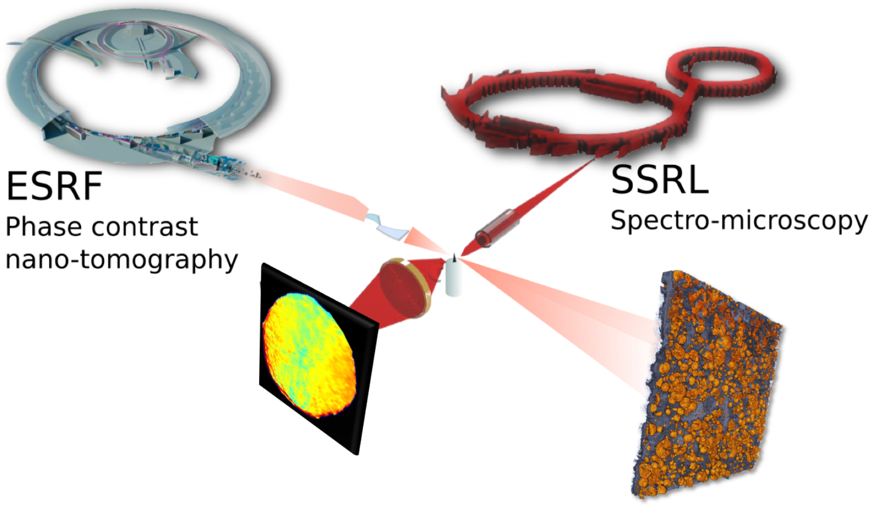

Supplementary Figure 6. Schematic illustration of correlative imaging using both the phase contrast nano-tomography at the ESRF and the spectro-microscopy at the SSRL. A unique particle was imaged using both instruments. To facilitate such measurement, a small piece of NMC cathode electrode was mounted on top of a Huber pin, which is compatible with both instruments.

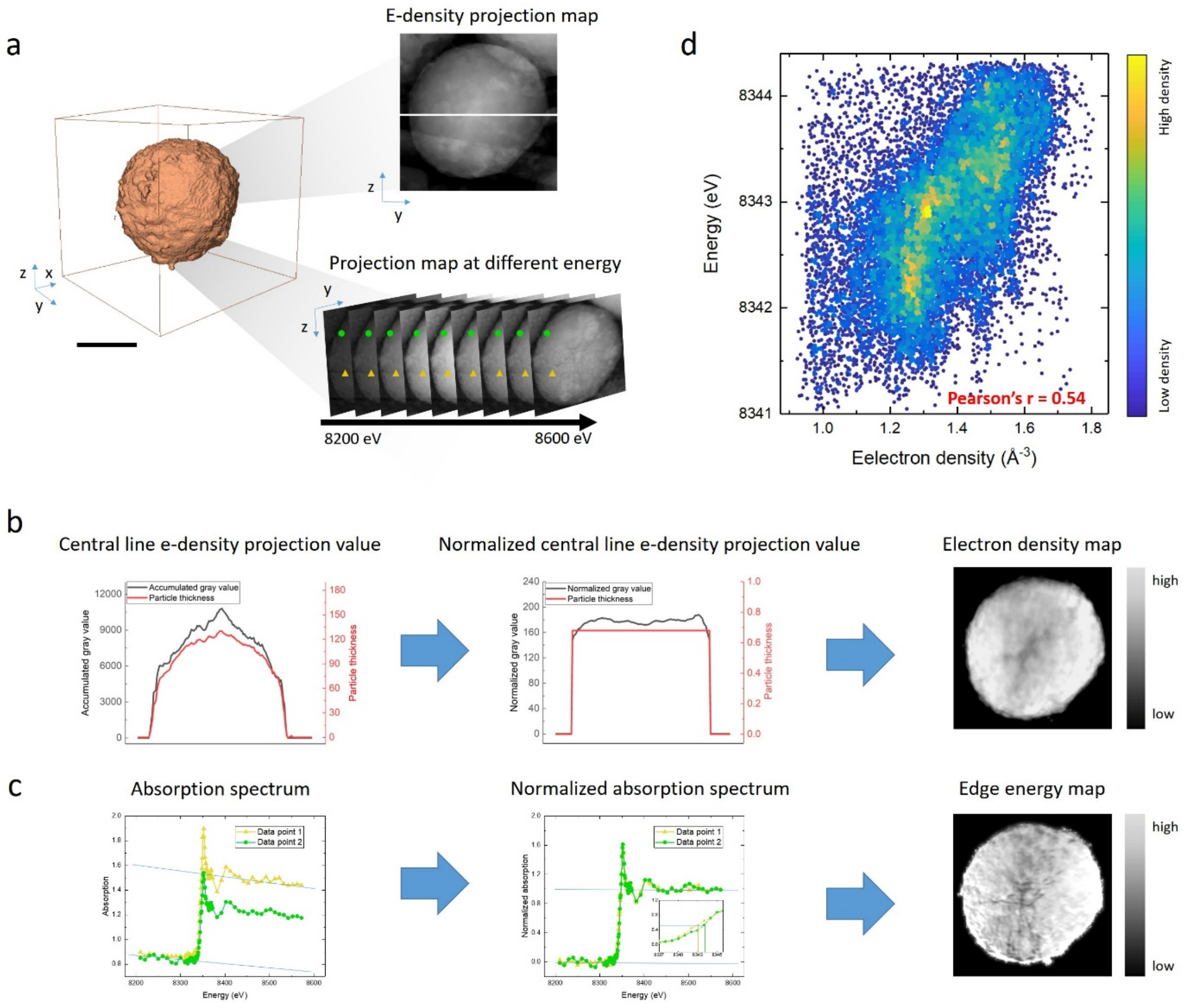

Supplementary Figure 7. Schematic illustration of the data normalization procedure for calculating the projective maps of electron density and the Ni valence state. (a) illustrates the particle that was scanned using both phase contrast nano-tomography and the spectro-microscopy. (b) shows the normalization procedure for the electron density map, which takes away the thickness information from the projective data. (c) shows spectroscopic normalization procedure for the Ni valence map. The correlation between the electron density and the Ni edge energy is shown in (d). A reasonably good degree of similarity (Pearson correlation coefficient equals 0.54) is observed, confirming the positive correlation between the electron density and the Ni valence state and, thus, the local SoC. The scale bar in (a) is 8 μm.

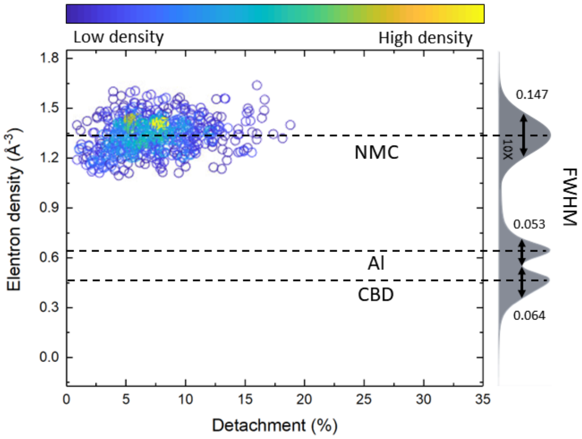

Supplementary Figure 8. Correlation plot of all the particles' electron density versus their respective degree of detachment from the CBD. The intensity variation in the phase contrast reconstruction result for the Al current collector, CBD, and the NMC811 particles are shown on the right of the plot. The FWHMs of the Al and CBD are quantified to be around 0.053 and 0.064, respectively. These two values are likely related with the actual measurement accuracy as one wouldn't anticipate density variation in Al. The FWHM of the NMC, on the other hand, is significantly larger (at ~0.147). This is likely due to the NMC's charge heterogeneity and the fine porosity that is beyond the resolution limit of the x-ray phase contrast tomography

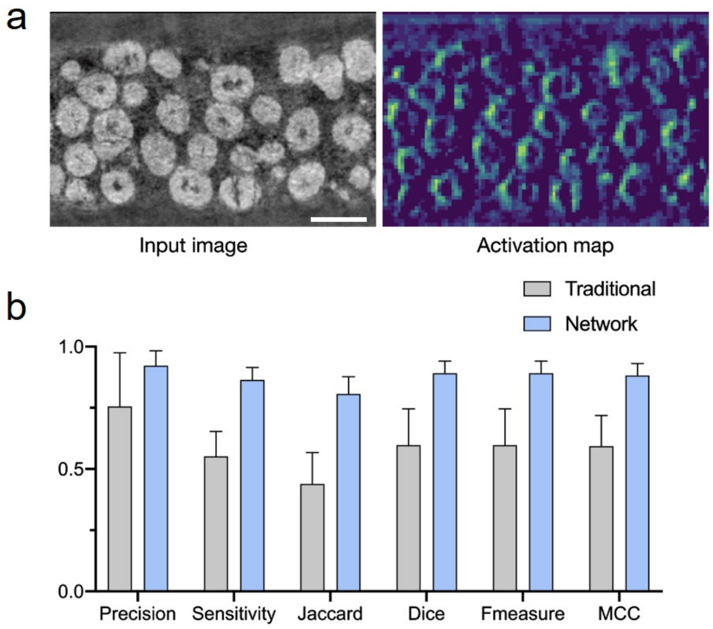

Supplementary Figure 9. Active map of neural network and performance comparison between traditional and machine learning method. (a) The input image and its corresponding activation map by the network, which highlights the particles' external boundaries. The emphasis of the particles' external boundaries with simultaneous suppression of the crack surface is exactly the desired functionality of the auto segmentation algorithm. (b) Performance comparison in terms of mean values (the error bars are the corresponding standard deviations) over all validation images between the machine-learning neural network method and the traditional watershed algorithm with respect to six typical evaluation metrics. It is evident that our approach significantly outperforms the conventional method in all of these aspects. The scale bar in (a) is 25 μm.

Supplementary Note 1:

The proposed NMC particle segmentation method consists of procedures for training and inference. In total, 221 nano-tomographic slices of NMC composite electrodes are manually labeled. These human-labeled images are treated as the ground truth. Among them, 155 images were used for training and validating the model and 66 images were held out for testing. Additional data augmentation step (random cropping, flipping, rotation and image scaling) was taken to increase the diversity of data available for training models, without actually collecting new data. The trained machine-learning model was then applied to the current dataset of monolayer NMC electrode. Note that the identification and segmentation step was done for each slice separately, and the 3D particles were reconstructed by linking the slices at different depths of the volume through the Hungarian maximum matching algorithm.

The Mask R-CNN model we are using in this study relies on a region proposals which are generated via a region proposal network. It can be split up to four parts: 1) scan the images and extract features with feature pyramid network; 2) find interested regions at different scales; 3) detect and classify the targets; 4) generate instance masks for each particle. Suplementary Figure 9a shows an example of the activation map during the detection process, which highlights the the particle-specific regions of the image. This activation map benefits the classification of each particle since the instance information is difficult to obtain directly from the intensities of the image. The binary cross-entropy loss function was used which measures how far away from the ground truth classes the prediction is for each of the particles. In total we train for 150 epochs using stochastic gradient descent with momentum of 0.9, starting with a learning rate of 0.001 and ending with a learning rate of 0.0001.

Six typical evaluation metrics were used to assess the performance of detecting corrected segmented particles. They are Precision (a.k.a positive predictive value), Sensitivity (a.k.a recall), Jaccard, Dice, F measure and MCC (Matthews correlation coefficient). The results were compared with the traditional watershed algorithm and showed significant improvement. More specifically, the marker-controlled watershed segmentation was used, and the intensities were used to generate marker locations. Parameters were tuned to get the best prediction results. Overall, the machine-learning model results in substantially better identification and segmentation of NMC particles. For instance, the trained model achieved precision and sensitivity of $92.2\% \pm 6\%$ and $86.4\% \pm 5\%$

on the validation dataset, while the traditional watershed algorithm gets $75.5\% \pm 22\%$ and $55.2\% \pm 10\%$. The results are summarized in Supplementary Figure 9b.
