# Supplementary material for: Machine-learning-revealed statistics of the particle-carbon/binder detachment in lithium-ion battery cathodes
Source: Nat Commun. 2020 May 8;11:2310. doi: 10.1038/s41467-020-16233-5 (PMC7210251; doi:10.1038/s41467-020-16233-5)
Supplement: Supplementary file 2 — Peer Review File [file 41467_2020_16233_MOESM2_ESM.pdf]

Reviewers' comments:

Reviewer #1 (Remarks to the Author):

This manuscript reports a mechanism study of particles' evolving (de)attachment with the conductive matrix in Li-ion battery cathodes based on both multiscale experimental approaches and machine-learning-assisted statistical analysis. The approach and the characterization are very nice and novel. As a result, the manuscript can be recommended for publication after the following suggestions are taken into account.

Foremost, why traditional methods fail to distinguish different particles and the machine learning approach demonstrates significantly improved performance? It would be better if the author can give more details about the difference between the ML approach and the traditional methods.

Second, the author talks about a very novel and important topic in this study, in contrast to the tremendous efforts in researching the active materials. So it would be better if the author can give some implications for follow-up experimental and computational study.

Third, in introduction section, the abbreviation, CBD, PDF-CT et al. should be explained.

Reviewer #2 (Remarks to the Author):

Jiang et al. reported a study of NMC-811 electrode system using hard x-ray nano-tomography based on the phase contrast modality. The resolution of their method is high, allowing them to identify CBD, NMC and void space. The authors connected the level of detachment of the NMC particle to relative electrical resistance using a numerical model. They then developed a machine learning method to automatically segment 650 electrode and statistically quantify how the level of detachment varies as a function C-rate and particle size. The authors also discussed a correlation between the local electron density and SoC.

High resolution characterisation of electrode structure subjected to different cycling conditions contributes towards understanding degradation, which is a very timely area of research. The authors' approach of high throughput segmentation and arriving at statistically meaningful conclusions is well-taken. I think this manuscript is potentially publishable in Nature Communication, after substantial revision.

My main concern is how the authors analysed their machine learning method. "Machine learning" are literally the first 2 words in the title, yet their description and discussion of the actual method is lacking. The authors mentioned "more details about the training procedure, as well as performance evaluation, will be described in a follow-up manuscript focusing on the computational aspect of this work", but this is inadequate.

Specific questions:

1. The authors should clearly state and discuss the size of the training set.

650 samples is a small dataset size for image recognition models. If the training set size is larger than 650, and the training set is hand annotated, then machine learning confers no time/performance advantage over hand annotation. In that case, the machine learning part in the manuscript is irrelevant.

2. How did the authors converge to this specific model? Which model architectures were considered?

What is the model accuracy on a held-out test set? Just showing handpicked results, e.g. Fig 3, is not informative.

3. What is "traditional segmentation" (c.f. Fig 3), and how does that compare with the authors' ML model?

4. How sensitive is the result presented in Fig 4 to the choice of ML algorithm to segment the images?

5. Data availability "upon reasonable request" is rather disappointing. Machine learning models tend to be quite intricate and essentially not reproducible unless the code and data are published in full. The authors should consider putting their code on GitHub and publish the data in a publicly accessible repository.

Reviewer #3 (Remarks to the Author):

The paper describes efforts to better understand degradation phenomena in LIB cathodes that are of nickel rich composition such as NMC811. In particular, it describes a method how data from X-ray phase contrast tomography and X-ray spectro-microscopy can subjected to a statical analysis based on machine learning. The experiments described are based on two battery cells and 650 particles analysed. Partial detachment is found of active particles from the particle ensemble embedded in carbon and binder. This reduces the electronic conductivity since only point contacts are left while the gaps created becomes filled with electrolyte solution which creates a larger solid-liquid interface.
The approach is of using X-ray tomography and X-ray spectro-microscopy together with machine learning to more precisely conduct the statistical analysis. While this combination of techniques appears very powerful the extraction of understanding of the cathode processes stays limited. The authors don't discuss the implications of the described processes or observations, e.g.
(i) In the inital phase of particles becoming detached the battery processes should be enhanced since the particle-electrolyte interface becomes larger. Since the charge carrier concentration is much higher in the solid state compared to the liquid an overall increased activity should be observed. Only in a later stage the contribution of the detaching particle should become deminished.
(ii) While the electron density changes with the SoC the authors rightly correlate it with the valence state of Ni. Here it would be useful to give an estimate of the valence change in Fig.5d and/or Fig.5e. In addition, it should be discussed why Mn and Co are not considered to undergo valency changes.
(iii) As stated, 10 charge-discharge cycles were performed at different C-rates. This may considered to be just in the break-in phase, longer cycling would be preferable.
(iii) All experiments are done ex-situ after disassembling the battery. In addition to saying that in-operando experiments are desirable, it should clearly be discussed where the limitation are with the current procedure and why in-operando experiments would be more revealing.
The paper is initial work illustrating a novel methodological approach but little discussion of what we learn from it regarding the battery processes. The paper is interesting from an experimental point of view, the scientific content is rather shallow. The paper should not be published in its current form.
A minor point: Why is Fig.1 in gray scale in contrast to all other figures, it is hard to make out the differences.

Point-to-point revision summary for manuscript NCOMMS-20-00106

TITLE: Machine-Learning-Revealed Statistics of the Particle-Carbon/Binder Detachment in Li-Ion Battery Cathodes

Reviewer #1:

This manuscript reports a mechanism study of particles' evolving (de)attachment with the conductive matrix in Li-ion battery cathodes based on both multiscale experimental approaches and machine-learning-assisted statistical analysis. The approach and the characterization are very nice and novel. As a result, the manuscript can be recommended for publication after the following suggestions are taken into account.

Response: We are grateful to reviewer #1 for his/her high-level comments on our work, which are very positive and encouraging. We also appreciate the specific suggestions and concerns raised by reviewer 1#. We have studied the reviewer's comments and carefully addressed them. We believe that the revisions have strengthened our manuscript significantly.

Foremost, why traditional methods fail to distinguish different particles and the machine learning approach demonstrates significantly improved performance? It would be better if the author can give more details about the difference between the ML approach and the traditional methods.

Response: We agree that, in addition to the direct comparison of the segmentation results, it will be useful to provide a narrative at the high-level. And we thank the reviewer for pointing this out.

The traditional watershed algorithm relies on the inner distance map as marking function and easily causes over-segmentation and/or under-segmentation when the boundaries of NMC particles are not clear or the signal-to-noise ratio of the image is low. More importantly, the particle's external boundary (versus the surface of the internal pores and cracks) cannot be easily defined in the conventional approach that is simply based on the local pixel intensity. Therefore, the formation of cracks in the particles (low-intensity features) could significantly and falsely alter the inner distance map and such an effect cannot be addressed by improving the image quality. As a result, due to the mechanical disintegration of the NMC particles, the traditional algorithm often mistakenly splits one particle into several parts (see Figure 3) with different labels assigned.

Our implementation of the Mask R-CNN method leverages on the existing machine learning model that is trained on the large-scale ImageNet dataset (a widely used on-line database for benchmarking of the object detection algorithms). Intuitively, this model takes advantage of the inherent hierarchical and multi-scale characteristic of a convolutional neural network to derive useful features for object detection. Starting from the pre-training weights, we optimize the network by incorporating the information of the NMC particle shape as defined in the manually annotated data. Such an approach effectively adds additional constraints in the segmentation and reinforces the overall quasi-spherical shape of the particles. Our results suggest that this approach shows significantly improved robustness against the formation of the inner-particle cracks, which would otherwise result in the identification of smaller irregularly shaped parts. For better illustration of this statement, we show in Figure S9a the comparison of the input image

and the activation map, which is extracted from an intermediate layer of our network. We point out here that only the particles' external boundaries are highlighted. The emphasis of the particles' external boundaries with simultaneous suppression of the crack surface is exactly the desired functionality of the auto segmentation algorithm and it is not possible to achieve such a purpose purely based on the intensity values of the input image.

For a more quantitative comparison of the results from the conventional watershed segmentation and the herein developed Mask R-CNN algorithm, we show in Figure S9b six different evaluation metrics. The detailed description of these evaluation metrics is included in the revised supplementary information. It is evident that our approach significantly outperforms the conventional method in all of these aspects.

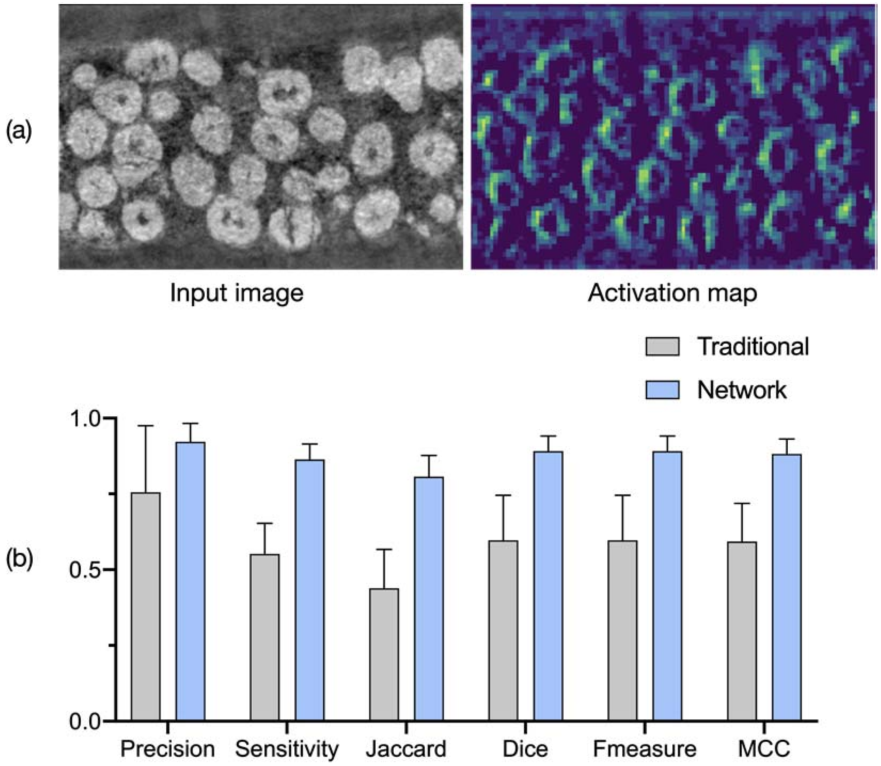

Figure S9. (a) The input image and its corresponding activation map by the network, which highlights the particle-specific regions of the image. (b) Performance comparison between the machine-learning neural network method and the traditional watershed algorithm with respect to six typical evaluation metrics

Second, the author talks about a very novel and important topic in this study, in contrast to the tremendous efforts in researching the active materials. So it would be better if the author can give some implications for follow-up experimental and computational study.

Response: We very much appreciate this forward-looking comment. The presented development of automatic segmentation and local resistance modeling capability set the basis for a number of follow-up studies.

For example, our segmentation approach could take the human out of the loop in analyzing a massive amount of data, and subsequently, could facilitate more sophisticated statistical analysis including the correlations of many different morphological characteristics and the chemomechanical breakdown of the particles. We could systematically evaluate the particle-size-dependence, the sphericity-dependence, the porosity-dependence, the particle-to-particle interaction, to name a few. The importance of this research direction is caused by the intrinsic complexity in the morphology-performance relationship. Such a complicated effect relies on a thorough analysis with statistical significance.

Another frontier challenge is to image and analyze these battery particles under operating conditions. The capability of tracking the same particles in real-time in an operando experiment could potentially open vast scientific opportunities by capturing metastable processes that only exist in nonequilibrium conditions. The in-situ experiment could also avoid the uncertainty caused by the cell to cell variation.

As a follow-up computational study, we are working on building a prediction model to detect the mechanical weak point in the composite electrode. Such a model is valuable to real-life battery operation and will be validated using experimental observations of the batteries that are subjected to different cycling rates and cut-off voltages.

Third, in introduction section, the abbreviation, CBD, PDF-CT et al. should be explained.

Response: We have added explanations of XRD and PDF-CT with proper references cited for readers who are interested in these methods.

Reviewer #2:

Jiang et al. reported a study of NMC-811 electrode system using hard x-ray nano-tomography based on the phase contrast modality. The resolution of their method is high, allowing them to identify CBD, NMC and void space. The authors connected the level of detachment of the NMC particle to relative electrical resistance using a numerical model. They then developed a machine learning method to automatically segment 650 electrode and statistically quantify how the level of detachment varies as a function C-rate and particle size. The authors also discussed a correlation between the local electron density and SoC.

High resolution characterisation of electrode structure subjected to different cycling conditions contributes towards understanding degradation, which is a very timely area of research. The authors' approach of high throughput segmentation and arriving at statistically meaningful conclusions is well-taken. I think this manuscript is potentially publishable in Nature Communication, after substantial revision.

Response: We sincerely thank Reviewer #2 for his/her assessments of our work. We are delighted to read that our work interests the reviewer. We also echo with the reviewer that the statistical analysis is of significant importance.

My main concern is how the authors analysed their machine learning method. "Machine learning" are literally the first 2 words in the title, yet their description and discussion of the actual method is lacking. The authors mentioned "more details about the training procedure, as well as performance evaluation, will be described in a follow-up manuscript focusing on the computational aspect of this work", but this is inadequate.

Response: We agree with the reviewer that a more detailed description of the machine learning method will be valuable to include in this manuscript. We address this question in three different aspects: 1) we added a high-level comparison of the conventional watershed segmentation method and our Mask R-CNN model; 2) we quantify the fidelity of the segmentation results using several different metrics in a systematic manner; 3) we made our source code and a test dataset freely available. Our colleagues in this field can further develop based on our existing effort. We address the specific questions in details below.

Specific questions:

1. The authors should clearly state and discuss the size of the training set.

650 samples is a small dataset size for image recognition models. If the training set size is larger than 650, and the training set is hand annotated, then machine learning confers no time/performance advantage over hand annotation. In that case, the machine learning part in the manuscript is irrelevant.

Response: In total, 221 nano-tomographic slices of NMC composite electrodes were manually labeled. These human-labeled images are treated as the ground truth. Among them, 155 images were used as a training dataset and the other 66 images were held out for validation. Additional data augmentation step (random cropping, flipping, rotation, and image scaling) was taken to increase the diversity of data available for training models, without actually collecting new data. The testing dataset has more than 1100 slices, which is substantially larger than the training set.

We would also point out that, with our development, the current network can be further optimized when a new dataset comes in. The re-optimization process of the algorithm requires only a very small amount of training data.

We have added these discussions in the revised supplementary information.

2. How did the authors converge to this specific model? Which model architectures were considered? What is the model accuracy on a held-out test set? Just showing handpicked results, e.g. Fig 3, is not informative.

Response: Our developments leverage on existing methods that are popular in the field of object detection. The chosen Mask R-CNN model is one of the state-of-the-art approaches. It has demonstrated outstanding performance in handling natural and biomedical images.

Rather than training the network end-to-end from the start, the ResNet-101 feature pyramid network was used and the model was initialized by the weights obtained from the large-scale ImageNet dataset (a widely used on-line database for benchmarking of the object detection algorithms). This transfer-learning-based machine-learning model was then fine-tuned using our human-labeled training data by incorporating the information of the NMC particle shape. Such an approach effectively adds additional constraint in the segmentation and reinforces the overall quasi-spherical shape of the particles. A schematic illustration of the machine learning model architecture is presented in Figure S4.

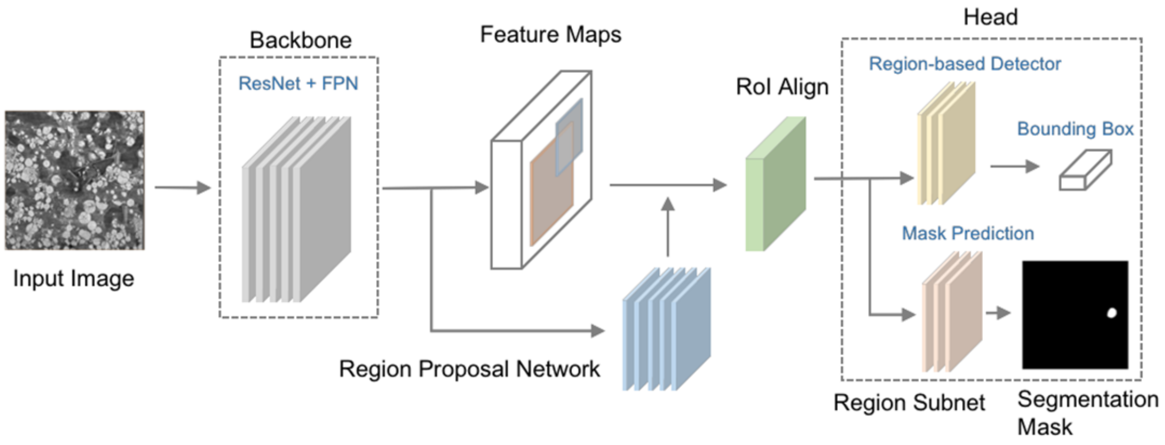

Figure S4. Schematic illustration of the herein developed machine learning model based on the Mask R-CNN for particle identification and segmentation. The model facilitates the detection of over 650 active particles in our phase contrast tomographic result, which set the basis for our statistical analysis. For the input slice, the residual neural network (ResNet) and feature pyramid network (FPN) are utilized as the backbone for feature extraction at different scales. After alignment of region-of-interest (RoI) with the extracted features, the head sub-network predicts bounding boxes for particles and then segments the particle inside the predicted boxes as a binary mask.

Regarding the held-out test, we used 66 out of 221 images for validation of our model. We present the held-out test results and the comparison versus the conventional segmentation results using six different evaluation metrics in Figure S9b. Our results suggest that this approach shows significantly improved robustness against the formation of the inner-particle cracks, which would otherwise result in the identification of smaller irregularly shaped parts.

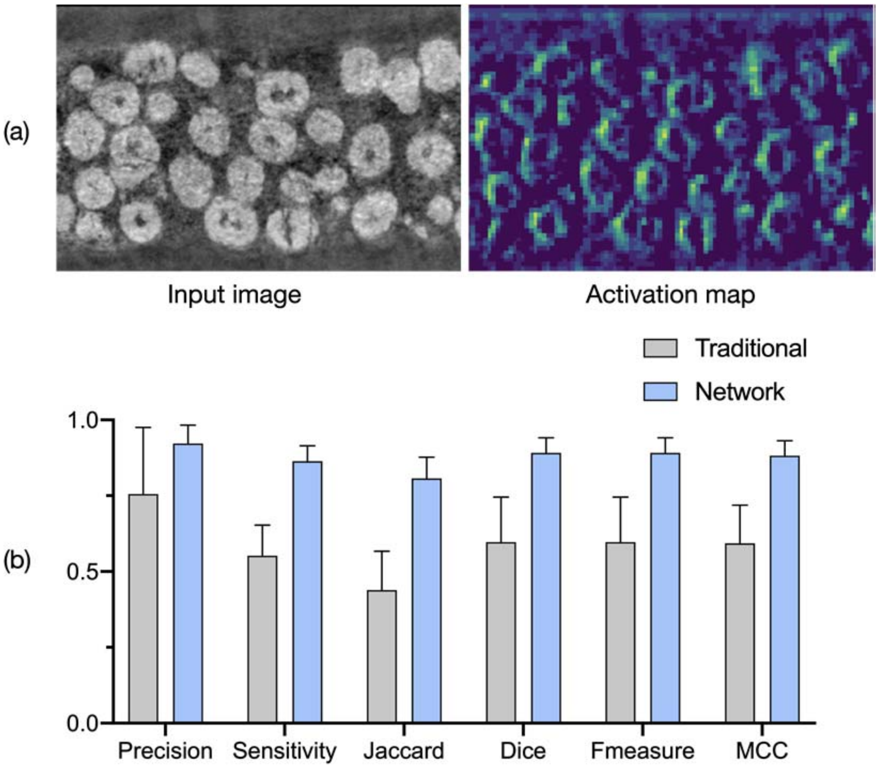

Figure S9. (a) The input image and its corresponding activation map by the network, which highlights the particle-specific regions of the image. (b) Performance comparison between the machine-learning neural network method and the traditional watershed algorithm with respect to six typical evaluation metrics.

3. What is "traditional segmentation" (c.f. Fig 3), and how does that compare with the authors' ML model?

Response: We compare our Mask R-CNN model to the watershed method, which is very broadly utilized in this field and is, therefore, referred to as the “traditional segmentation”.

The traditional watershed algorithm relies on the inner distance map as marking function and easily causes over-segmentation and/or under-segmentation when the boundaries of NMC particles are not clear or the signal-to-noise ratio of the image is low. More importantly, the particle’s external boundary (versus the surface of the pores and cracks) cannot be easily defined in the conventional approach that is simply based on the local pixel intensity. Therefore, the formation of cracks in the particles (low-intensity features) could significantly and falsely alter the inner distance map and such an effect cannot be addressed by improving the image quality. As a result, due to the mechanical disintegration of the NMC particles, the traditional algorithm often mistakenly splits one particle into several parts (see Figure 3) with different labels assigned.

Our implementation of the Mask R-CNN method leverages on the existing machine learning model that is trained on the large-scale ImageNet dataset (a widely used on-line database for benchmarking of the object detection algorithms). Intuitively, this model takes advantage of the inherent hierarchical and multi-scale characteristic of a convolutional neural network to derive

useful features for object detection. Starting from the pre-training weights, we optimize the network by incorporating the information of the NMC particle shape as defined in the manually annotated data. Such an approach effectively adds additional constraint in the segmentation and reinforces the overall quasi-spherical shape of the particles. Our results suggest that this approach shows significantly improved robustness against the formation of the inner-particle cracks, which would otherwise result in the identification of smaller irregularly shaped parts. For better illustration of this statement, we show in Figure S9a the comparison of the input image and the activation map, which is extracted from an intermediate layer of our network. We highlight here that only the particles' external boundaries are highlighted. The emphasis of the particles' external boundaries with simultaneous suppression of the crack surface is exactly the desired functionality of the auto segmentation algorithm and it is not possible to achieve such a purpose purely based on the intensity values of the input image.

For a more quantitative comparison of the results from the conventional watershed segmentation and the herein developed Mask R-CNN algorithm, we show in Figure S9b six different evaluation metrics. The detailed description of these evaluation metrics is included in the revised supplementary information. It is evident that our approach significantly outperforms the conventional method in all of these aspects.

4. How sensitive is the result presented in Fig 4 to the choice of ML algorithm to segment the images?

Response: The sensitivity comes from the accuracy of the segmentation. The observation in Figure 4 is based on the statistical analysis of a massive amount of segmented particles by our machine-learning method. Inaccurate segmentation would harm the accuracy of the probability distribution (Figure 4) in two different ways. Firstly, as mentioned above, the conventional watershed segmentation often mistakenly divides an individual particle into several pieces. Such an effect would dramatically and falsely change the statistics of the particle size distribution. Moreover, the evaluation of the particles' detachment from the conductive carbon and binder domain relies on the evaluation of the particle's exterior surface. Incorrect segmentation could falsely identify the particles' internal pore and crack as the exterior surface, on which the detachment evaluation is computed.

Regarding the potential choice of different ML algorithms, we are aware that there are other approaches that could also be applicable. While we have sufficient validation of the current approach, in the follow-up studies it will be useful to look into different options.

5. Data availability "upon reasonable request" is rather disappointing. Machine learning models tend to be quite intricate and essentially not reproducible unless the code and data are published in full. The authors should consider putting their code on GitHub and publish the data in a publicly accessible repository.

Response: We declare that we used the standard data availability statement of Nat. Comm. in our original submission. The source code and detailed instructions are now made publicly available at the GitHub repository: https://github.com/hijizhou/LIBNet. We welcome our colleagues to evaluate and to contribute to the further developments.

Note: this webpage will be published once our manuscript is on-line. This is because some of the text and figures in the instruction overlaps with our manuscript. The screenshots are included below and uploaded as a separate file for the review process.

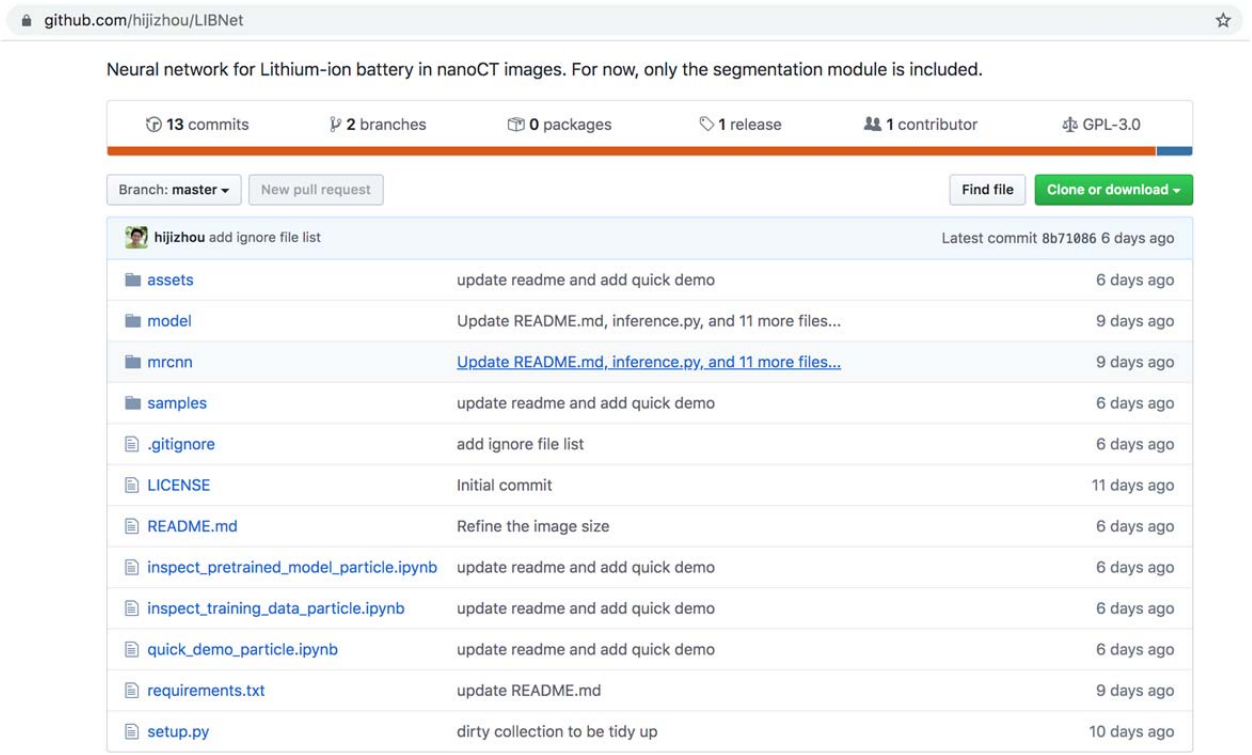

Reviewer #3:

The paper describes efforts to better understand degradation phenomena in LIB cathodes that are of nickel rich composition such as NMC811. In particular, it describes a method how data from X-ray phase contrast tomography and X-ray spectro-microscopy can subjected to a statical analysis based on machine learning. The experiments described are based on two battery cells and 650 particles analysed. Partial detachment is found of active particles from the particle ensemble embedded in carbon and binder. This reduces the electronic conductivity since only point contacts are left while the gaps created becomes filled with electrolyte solution which creates a larger solid-liquid interface.

The approach is of using X-ray tomography and X-ray spectro-microscopy together with machine learning to more precisely conduct the statistical analysis. While this combination of techniques appears very powerful the extraction of understanding of the cathode processes stays limited. The authors don't discuss the implications of the described processes or observations, e.g.

Response: We are grateful to reviewer #3 for his/her assessments on the technical aspects of our work. We also appreciate the specific suggestions and concerns raised by reviewer #3. We

have studied all the comments and suggestions and have revised our manuscript accordingly and substantially.

(i) In the inital phase of particles becoming detached the battery processes should be enhanced since the particle-electrolyte interface becomes larger. Since the charge carrier concentration is much higher in the solid state compared to the liquid an overall increased activity should be observed. Only in a later stage the contribution of the detaching particle should become deminished.

Response:

We agree with the reviewer that the battery performance enhancement could be the case in the early stage of the particle detachment from the CBD and the reason is in-line with the description by the reviewer.

To illustrate such an effect, we construct a conceptual model to describe the influence of particle detachment on the local conductivity of electron and Li-ion, which is shown in the revised Figure 4e. As discussed in the manuscript, the particle detachment will rearrange the local electrical conductivity and favor the local ionic conductivity as it leads to better contact between the liquid electrolyte and the NMC particle. The competing factors (enhanced ionic conductivity and reduced electrical conductivity) collectively govern the particle's behavior. A particle's actual contribution to the cell level chemistry is dominated by whichever is worse. As a consequence, the performance of the particle could slightly improve in the early stage of the particle detachment due to the improved ionic conductivity. In more severely detached circumstances, the decreased electrical conductivity takes over and results in an overall negative impact.

We add these discussions to the manuscript and changed Figure 4. We also declare here that a more systematic study on such an effect is very valuable. Ideally one would do such evaluation under operating conditions. The herein developed automatic segmentation and quantification methods can well be adapted in the follow-up investigations.

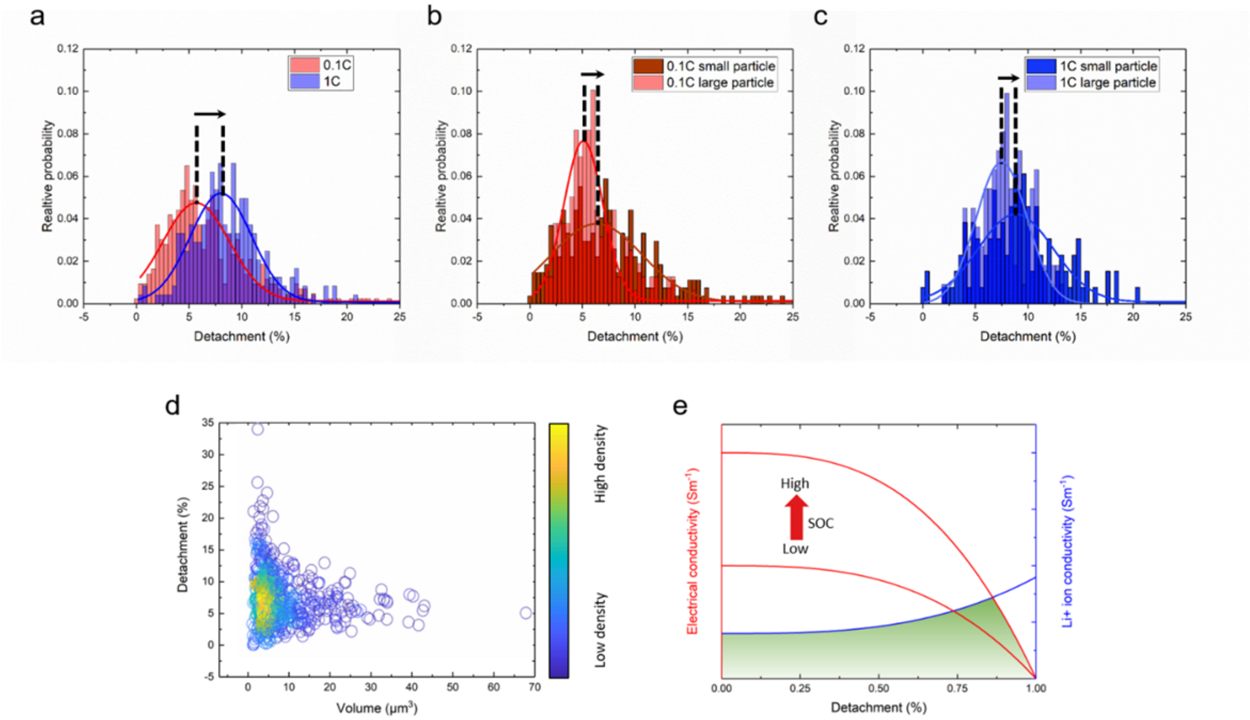

Figure 4. Statistical comparison of the degree of particle detachment from the CBD matrix as a function of cycling rate (panel (a)) and particle size (panels (b) and (c)), respectively. The degree of particle detachment is also plotted against the respective particle volume in panel (d) for all the 650 NMC particles studied in this work. Panel (e) illustrates, schematically, the changes of the electrical (red) and ionic (blue) conductivity as a function of the particle detachment. The red curve moves up and down upon cycling as a function of the SoC. A particle's actual contribution to the cell level chemistry is affected by both the conductivities of the electrons and Li+ ions and is dominated by whichever is worse. The particle's actual contribution falls in the green shaded area.

(ii) While the electron density changes with the SoC the authors rightly correlate it with the valence state of Ni. Here it would be useful to give an estimate of the valence change in Fig.5d and/or Fig.5e. In addition, it should be discussed why Mn and Co are not considered to undergo valency changes.

Response: We are grateful for the reviewer's comment. We have benchmarked the color scale of Ni valence map in Figure 5d quantitatively. We conducted this benchmarking by comparing the spectra of the scanned NMC particle to that of the known standards. As shown in Figure R1, the K-edge energy of the Ni XANES spectra positively correlates with the Ni's valence state. Therefore, the Ni's K-edge energy has been broadly utilized as a key descriptor for interpretation of the Ni XANES data.

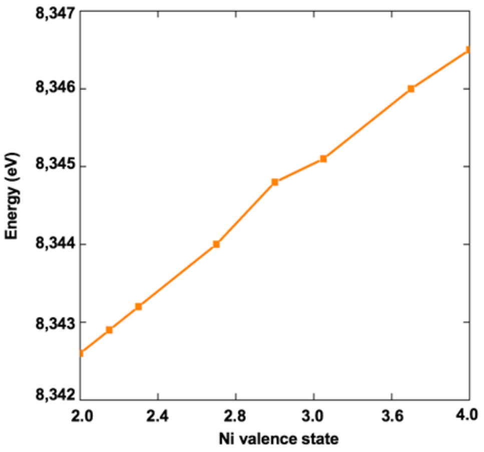

Figure R1. Correlation of the Ni K-edge energy and the Ni's valence state. Data retrieved from Kuppan et al., Nature Communications 8, 14309 (2017).

For NMC811 cathode material, the valence state of Ni cations shows dramatic changes during the charging/discharging process. The Mn^{4+} usually remains electrochemically inactive during charging/discharging and the Co cations only partially contribute to the charge compensation, due to the partial oxidization of Co^{3+} to Co^{4+} and the low Co content in NMC811 material. It has been demonstrated in the literature that the changes in Co XANES are insignificant. [Kim et al., J. Power Sources 2011, 196, 6710−6715; Sun et al., J. Electrochem. Soc. 2011, 159, A1-A5; Tian et al., J. Electrochem. Soc. 2018, 165, A696-A704].

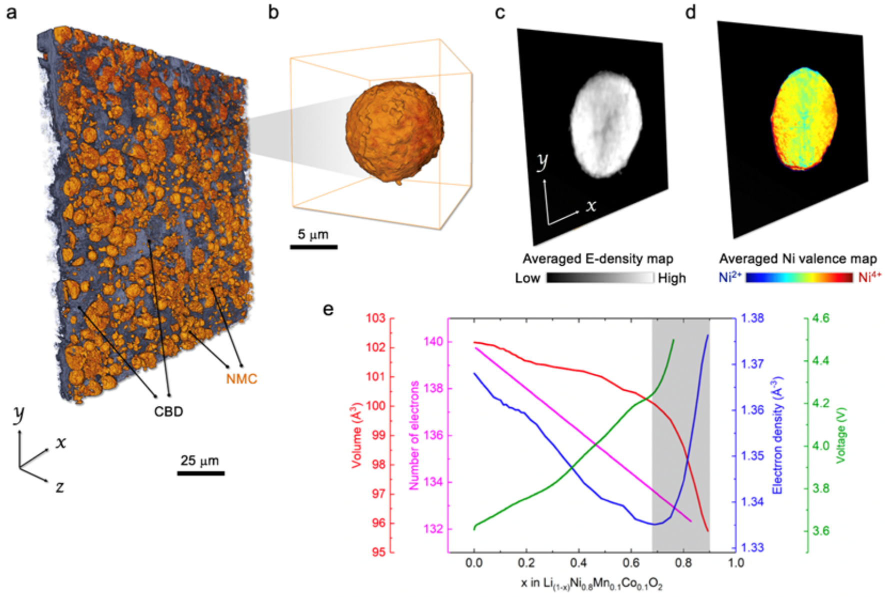

Figure 5. The local electron density as a proxy for the local state of charge. Panel (a) is the 3D rendering of an electrode with a monolayer of NMC 811 particles. Panels (b) to (d) show our correlative phase contrast (panel (c), collected at ID16A, ESRF, Grenoble, France) and

spectroscopic (panel (d), collected at 6-2C, SSRL, Menlo Park, California, USA) x-ray imaging on the same NMC secondary particle (panel (b)). (c) and (d) show a reasonable similarity. Panel (e) shows the changes of lattice unit cell volume, number of electrons per lattice unit cell, voltage (1C) and electron density as a function of the SoC. The scale bars in (a) and (b) are 25 µm and 5 µm, respectively.

(iii) As stated, 10 charge-discharge cycles were performed at different C-rates. This may considered to be just in the break-in phase, longer cycling would be preferable.

Response: We agree with the reviewer about this. Our statistical analysis of the particle detachment shows that the damage in our sample is still at a relatively low level. While it is desirable to look into the battery electrode that has gone through extensive cycling, we would highlight that our on-going effort to conduct this type of measurements under in-situ conditions, which would eliminate the cell-to-cell variation and facilitate the tracking of the same particles over long term cycling. Our established data analysis tools will play an important role in follow-up studies. Another point we would like to make is that it is very desirable to detect or even to predict the mechanical failure point in the composite electrode in the early state. For the development of this predictive model, it is important to acquire the high-resolution tomography data in both the early cycles and the late cycles.

(iii) All experiments are done ex-situ after disassembling the battery. In addition to saying that in-operando experiments are desirable, it should clearly be discussed where the limitation are with the current procedure and why in-operando experiments would be more revealing.

Response: There are few limitations with the current procedure. First of all, the disassembling process could cause damage to the electrode and, subsequently, affect the result of the statistical analysis. Second, the relaxation of the electrode may lead to charge redistribution in the electrode, making it difficult to evaluate the electrode scale chemical heterogeneity, which could be thermodynamically metastable. Finally, the cell-to-cell discrepancy is a common effect, which could add more complexity to the analysis and interpretation. All these limitations can be tackled by implementing an operando experimental strategy. We are actively pursuing this and our developments in the computational aspects (this paper) will be very useful in the follow-up studies.

The paper is initial work illustrating a novel methodological approach but little discussion of what we learn from it regarding the battery processes. The paper is interesting from an experimental point of view, the scientific content is rather shallow. The paper should not be published in its current form.

Response: We are grateful for the constructive comments. We wish that our substantial revisions of the manuscript have addressed the concerns from the reviewer #3.

A minor point: Why is Fig.1 in gray scale in contrast to all other figures, it is hard to make out the differences.

Response: It is useful to present the imaging data in its most native form in Figure 1. The color-coded rendering of the data relies on accurate segmentation. The superior resolution and image contrast offered by the phase contrast nano-tomography technique lays out the basis for the presented development of ML and modeling. It is our intention to communicate this point by presenting the tomography data without too many cosmetic decorations.

REVIEWERS' COMMENTS:

Reviewer #2 (Remarks to the Author):

The authors have addressed my comments by substantially deepening the discussion of their machine learning algorithm. They have also pledged to make the code and data openly available. I think the results presented in the manuscript are novel and key additions to the field. I recommend publication in Nature Communications.

Point-to-point revision summary for manuscript NCOMMS-20-00106

TITLE: Machine-Learning-Revealed Statistics of the Particle-Carbon/Binder Detachment in Li-Ion Battery Cathodes

Reviewer #1:

This manuscript reports a mechanism study of particles' evolving (de)attachment with the conductive matrix in Li-ion battery cathodes based on both multiscale experimental approaches and machine-learning-assisted statistical analysis. The approach and the characterization are very nice and novel. As a result, the manuscript can be recommended for publication after the following suggestions are taken into account.

Response: We are grateful to reviewer #1 for his/her high-level comments on our work, which are very positive and encouraging. We also appreciate the specific suggestions and concerns raised by reviewer 1#. We have studied the reviewer's comments and carefully addressed them. We believe that the revisions have strengthened our manuscript significantly.

Foremost, why traditional methods fail to distinguish different particles and the machine learning approach demonstrates significantly improved performance? It would be better if the author can give more details about the difference between the ML approach and the traditional methods.

Response: We agree that, in addition to the direct comparison of the segmentation results, it will be useful to provide a narrative at the high-level. And we thank the reviewer for pointing this out.

The traditional watershed algorithm relies on the inner distance map as marking function and easily causes over-segmentation and/or under-segmentation when the boundaries of NMC particles are not clear or the signal-to-noise ratio of the image is low. More importantly, the particle's external boundary (versus the surface of the internal pores and cracks) cannot be easily defined in the conventional approach that is simply based on the local pixel intensity. Therefore, the formation of cracks in the particles (low-intensity features) could significantly and falsely alter the inner distance map and such an effect cannot be addressed by improving the image quality. As a result, due to the mechanical disintegration of the NMC particles, the traditional algorithm often mistakenly splits one particle into several parts (see Figure 3) with different labels assigned.

Our implementation of the Mask R-CNN method leverages on the existing machine learning model that is trained on the large-scale ImageNet dataset (a widely used on-line database for benchmarking of the object detection algorithms). Intuitively, this model takes advantage of the inherent hierarchical and multi-scale characteristic of a convolutional neural network to derive useful features for object detection. Starting from the pre-training weights, we optimize the network by incorporating the information of the NMC particle shape as defined in the manually annotated data. Such an approach effectively adds additional constraints in the segmentation and reinforces the overall quasi-spherical shape of the particles. Our results suggest that this approach shows significantly improved robustness against the formation of the inner-particle cracks, which would otherwise result in the identification of smaller irregularly shaped parts. For better illustration of this statement, we show in Figure S9a the comparison of the input image

and the activation map, which is extracted from an intermediate layer of our network. We point out here that only the particles' external boundaries are highlighted. The emphasis of the particles' external boundaries with simultaneous suppression of the crack surface is exactly the desired functionality of the auto segmentation algorithm and it is not possible to achieve such a purpose purely based on the intensity values of the input image.

For a more quantitative comparison of the results from the conventional watershed segmentation and the herein developed Mask R-CNN algorithm, we show in Figure S9b six different evaluation metrics. The detailed description of these evaluation metrics is included in the revised supplementary information. It is evident that our approach significantly outperforms the conventional method in all of these aspects.

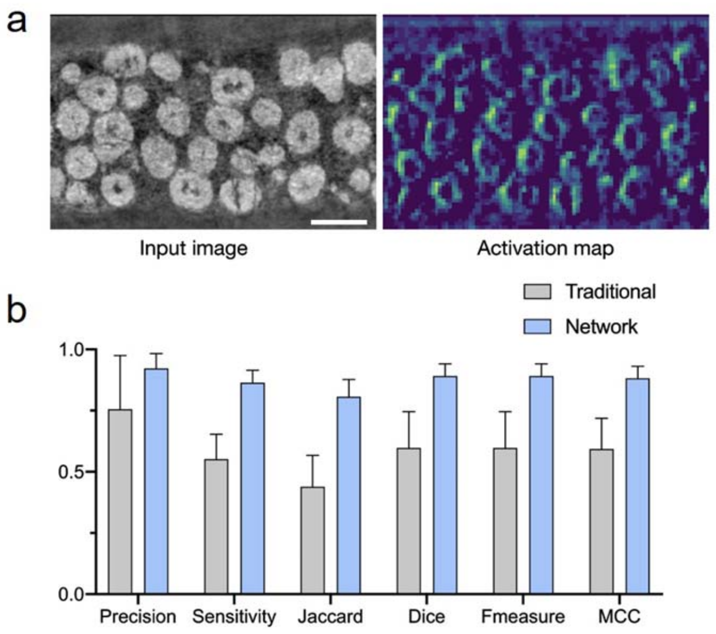

Supplementary Figure 9. Active map of neural network and performance comparison between traditional and machine learning method. (a) The input image and its corresponding activation map by the network, which highlights the particles' external boundaries. The emphasis of the particles' external boundaries with simultaneous suppression of the crack surface is exactly the desired functionality of the auto segmentation algorithm. (b) Performance comparison in terms of mean values and standard deviation (represented by error bars) over all validation images between the machine-learning neural network method and the traditional watershed algorithm with respect to six typical evaluation metrics. It is evident that our approach significantly outperforms the conventional method in all of these aspects. The scale bar in (a) is 25 μm.

Second, the author talks about a very novel and important topic in this study, in contrast to the tremendous efforts in researching the active materials. So it would be better if the author can give some implications for follow-up experimental and computational study.

Response: We very much appreciate this forward-looking comment. The presented development of automatic segmentation and local resistance modeling capability set the basis for a number of follow-up studies.

For example, our segmentation approach could take the human out of the loop in analyzing a massive amount of data, and subsequently, could facilitate more sophisticated statistical analysis including the correlations of many different morphological characteristics and the chemomechanical breakdown of the particles. We could systematically evaluate the particle-size-dependence, the sphericity-dependence, the porosity-dependence, the particle-to-particle interaction, to name a few. The importance of this research direction is caused by the intrinsic complexity in the morphology-performance relationship. Such a complicated effect relies on a thorough analysis with statistical significance.

Another frontier challenge is to image and analyze these battery particles under operating conditions. The capability of tracking the same particles in real-time in an operando experiment could potentially open vast scientific opportunities by capturing metastable processes that only exist in nonequilibrium conditions. The in-situ experiment could also avoid the uncertainty caused by the cell to cell variation.

As a follow-up computational study, we are working on building a prediction model to detect the mechanical weak point in the composite electrode. Such a model is valuable to real-life battery operation and will be validated using experimental observations of the batteries that are subjected to different cycling rates and cut-off voltages.

Third, in introduction section, the abbreviation, CBD, PDF-CT et al. should be explained.

Response: We have added explanations of XRD and PDF-CT with proper references cited for readers who are interested in these methods.

Reviewer #2:

Jiang et al. reported a study of NMC-811 electrode system using hard x-ray nano-tomography based on the phase contrast modality. The resolution of their method is high, allowing them to identify CBD, NMC and void space. The authors connected the level of detachment of the NMC particle to relative electrical resistance using a numerical model. They then developed a machine learning method to automatically segment 650 electrode and statistically quantify how the level of detachment varies as a function C-rate and particle size. The authors also discussed a correlation between the local electron density and SoC.

High resolution characterisation of electrode structure subjected to different cycling conditions contributes towards understanding degradation, which is a very timely area of research. The authors' approach of high throughput segmentation and arriving at statistically meaningful conclusions is well-taken. I think this manuscript is potentially publishable in Nature Communication, after substantial revision.

Response: We sincerely thank Reviewer #2 for his/her assessments of our work. We are delighted to read that our work interests the reviewer. We also echo with the reviewer that the statistical analysis is of significant importance.

My main concern is how the authors analysed their machine learning method. "Machine learning" are literally the first 2 words in the title, yet their description and discussion of the actual method is lacking. The authors mentioned "more details about the training procedure, as well as performance evaluation, will be described in a follow-up manuscript focusing on the computational aspect of this work", but this is inadequate.

Response: We agree with the reviewer that a more detailed description of the machine learning method will be valuable to include in this manuscript. We address this question in three different aspects: 1) we added a high-level comparison of the conventional watershed segmentation method and our Mask R-CNN model; 2) we quantify the fidelity of the segmentation results using several different metrics in a systematic manner; 3) we made our source code and a test dataset freely available. Our colleagues in this field can further develop based on our existing effort. We address the specific questions in details below.

Specific questions:

1. The authors should clearly state and discuss the size of the training set.

650 samples is a small dataset size for image recognition models. If the training set size is larger than 650, and the training set is hand annotated, then machine learning confers no time/performance advantage over hand annotation. In that case, the machine learning part in the manuscript is irrelevant.

Response: In total, 221 nano-tomographic slices of NMC composite electrodes were manually labeled. These human-labeled images are treated as the ground truth. Among them, 155 images were used as a training dataset and the other 66 images were held out for validation. Additional data augmentation step (random cropping, flipping, rotation, and image scaling) was taken to increase the diversity of data available for training models, without actually collecting new data. The testing dataset has more than 1100 slices, which is substantially larger than the training set.

We would also point out that, with our development, the current network can be further optimized when a new dataset comes in. The re-optimization process of the algorithm requires only a very small amount of training data.

We have added these discussions in the revised supplementary information.

2. How did the authors converge to this specific model? Which model architectures were considered? What is the model accuracy on a held-out test set? Just showing handpicked results, e.g. Fig 3, is not informative.

Response: Our developments leverage on existing methods that are popular in the field of object detection. The chosen Mask R-CNN model is one of the state-of-the-art approaches. It has demonstrated outstanding performance in handling natural and biomedical images.

Rather than training the network end-to-end from the start, the ResNet-101 feature pyramid network was used and the model was initialized by the weights obtained from the large-scale ImageNet dataset (a widely used on-line database for benchmarking of the object detection algorithms). This transfer-learning-based machine-learning model was then fine-tuned using our human-labeled training data by incorporating the information of the NMC particle shape. Such an approach effectively adds additional constraint in the segmentation and reinforces the overall quasi-spherical shape of the particles. A schematic illustration of the machine learning model architecture is presented in Supplementary Figure 4.

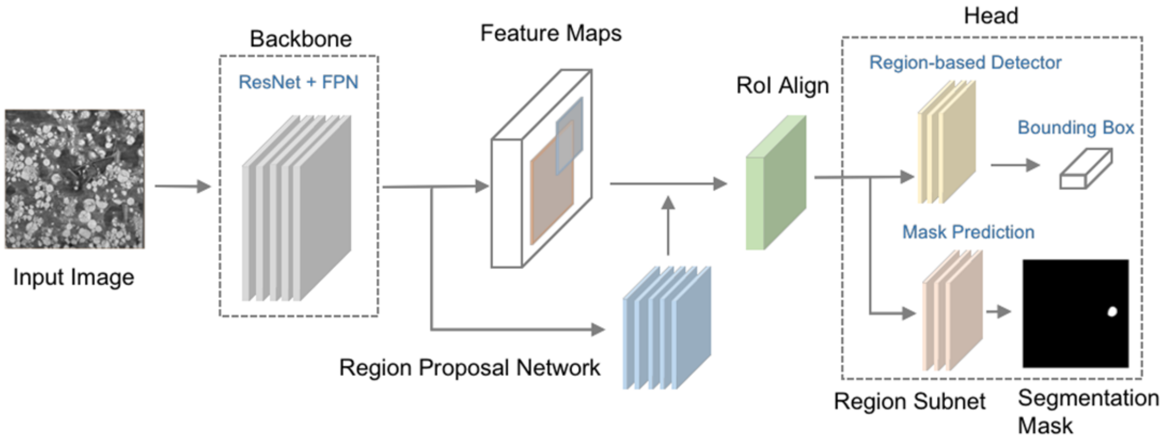

Supplementary Figure 4. Schematic illustration of the herein developed machine learning model based on the Mask R-CNN for particle identification and segmentation. The model facilitates the detection of over 650 active particles in our phase contrast tomographic result, which set the basis for our statistical analysis. For the input slice, the residual neural network (ResNet) and feature pyramid network (FPN) are utilized as the backbone for feature extraction at different scales. After alignment of region-of-interest (RoI) with the extracted features, the head sub-network predicts bounding boxes for particles and then segments the particle inside the predicted boxes as a binary mask.

Regarding the held-out test, we used 66 out of 221 images for validation of our model. We present the held-out test results and the comparison versus the conventional segmentation results using six different evaluation metrics in Figure S9b. Our results suggest that this approach shows significantly improved robustness against the formation of the inner-particle cracks, which would otherwise result in the identification of smaller irregularly shaped parts.

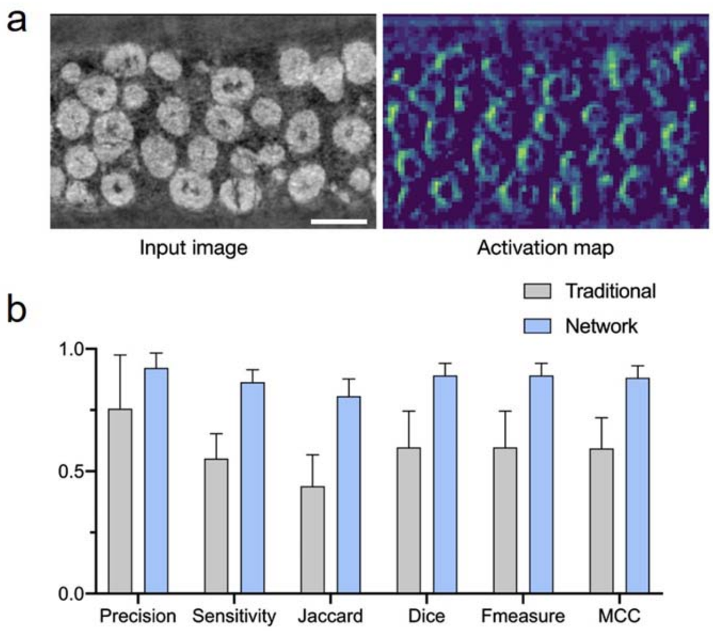

Supplementary Figure 9. Active map of neural network and performance comparison between traditional and machine learning method. (a) The input image and its corresponding activation map by the network, which highlights the particles' external boundaries. The emphasis of the particles' external boundaries with simultaneous suppression of the crack surface is exactly the desired functionality of the auto segmentation algorithm. (b) Performance comparison in terms of mean values and standard deviation (represented by error bars) over all validation images between the machine-learning neural network method and the traditional watershed algorithm with respect to six typical evaluation metrics. It is evident that our approach significantly outperforms the conventional method in all of these aspects. The scale bar in (a) is 25 μm.

3. What is "traditional segmentation" (c.f. Fig 3), and how does that compare with the authors' ML model?

Response: We compare our Mask R-CNN model to the watershed method, which is very broadly utilized in this field and is, therefore, referred to as the "traditional segmentation".

The traditional watershed algorithm relies on the inner distance map as marking function and easily causes over-segmentation and/or under-segmentation when the boundaries of NMC particles are not clear or the signal-to-noise ratio of the image is low. More importantly, the particle's external boundary (versus the surface of the pores and cracks) cannot be easily defined in the conventional approach that is simply based on the local pixel intensity. Therefore, the formation of cracks in the particles (low-intensity features) could significantly and falsely alter the inner distance map and such an effect cannot be addressed by improving the image quality. As a result, due to the mechanical disintegration of the NMC particles, the traditional algorithm often mistakenly splits one particle into several parts (see Figure 3) with different labels assigned.

Our implementation of the Mask R-CNN method leverages on the existing machine learning model that is trained on the large-scale ImageNet dataset (a widely used on-line database for benchmarking of the object detection algorithms). Intuitively, this model takes advantage of the inherent hierarchical and multi-scale characteristic of a convolutional neural network to derive useful features for object detection. Starting from the pre-training weights, we optimize the network by incorporating the information of the NMC particle shape as defined in the manually annotated data. Such an approach effectively adds additional constraint in the segmentation and reinforces the overall quasi-spherical shape of the particles. Our results suggest that this approach shows significantly improved robustness against the formation of the inner-particle cracks, which would otherwise result in the identification of smaller irregularly shaped parts. For better illustration of this statement, we show in Figure S9a the comparison of the input image and the activation map, which is extracted from an intermediate layer of our network. We highlight here that only the particles' external boundaries are highlighted. The emphasis of the particles' external boundaries with simultaneous suppression of the crack surface is exactly the desired functionality of the auto segmentation algorithm and it is not possible to achieve such a purpose purely based on the intensity values of the input image.

For a more quantitative comparison of the results from the conventional watershed segmentation and the herein developed Mask R-CNN algorithm, we show in Figure S9b six different evaluation metrics. The detailed description of these evaluation metrics is included in the revised supplementary information. It is evident that our approach significantly outperforms the conventional method in all of these aspects.

4. How sensitive is the result presented in Fig 4 to the choice of ML algorithm to segment the images?

Response: The sensitivity comes from the accuracy of the segmentation. The observation in Figure 4 is based on the statistical analysis of a massive amount of segmented particles by our machine-learning method. Inaccurate segmentation would harm the accuracy of the probability distribution (Figure 4) in two different ways. Firstly, as mentioned above, the conventional watershed segmentation often mistakenly divides an individual particle into several pieces. Such an effect would dramatically and falsely change the statistics of the particle size distribution. Moreover, the evaluation of the particles' detachment from the conductive carbon and binder domain relies on the evaluation of the particle's exterior surface. Incorrect segmentation could falsely identify the particles' internal pore and crack as the exterior surface, on which the detachment evaluation is computed.

Regarding the potential choice of different ML algorithms, we are aware that there are other approaches that could also be applicable. While we have sufficient validation of the current approach, in the follow-up studies it will be useful to look into different options.

5. Data availability "upon reasonable request" is rather disappointing. Machine learning models tend to be quite intricate and essentially not reproducible unless the code and data are published in full. The authors should consider putting their code on GitHub and publish the data in a publicly accessible repository.

Response: We declare that we used the standard data availability statement of Nat. Comm. in our original submission. The source code and detailed instructions are now made publicly

available at the GitHub repository: https://github.com/hijizhou/LIBNet. We welcome our colleagues to evaluate and to contribute to the further developments.

Note: this webpage will be published once our manuscript is on-line. This is because some of the text and figures in the instruction overlaps with our manuscript. The screenshots are included below and uploaded as a separate file for the review process.

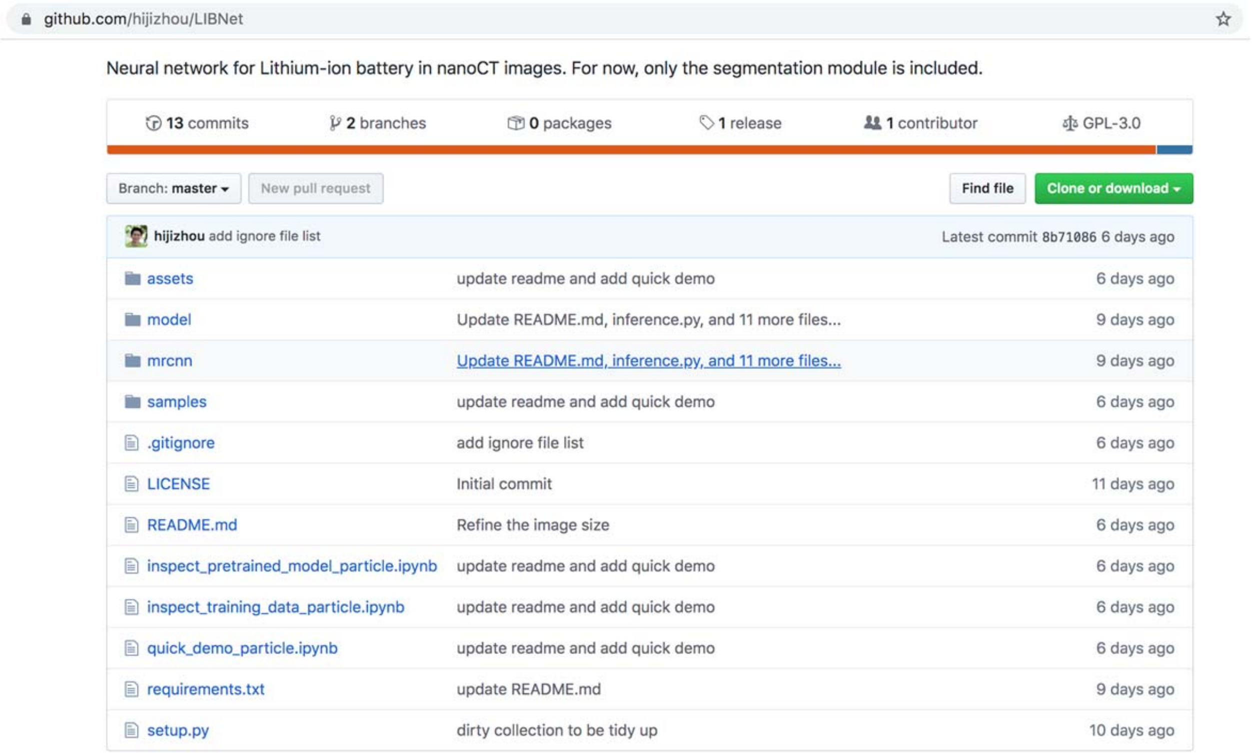

Reviewer #3:

The paper describes efforts to better understand degradation phenomena in LIB cathodes that are of nickel rich composition such as NMC811. In particular, it describes a method how data from X-ray phase contrast tomography and X-ray spectro-microscopy can subjected to a statical analysis based on machine learning. The experiments described are based on two battery cells and 650 particles analysed. Partial detachment is found of active particles from the particle ensemble embedded in carbon and binder. This reduces the electronic conductivity since only point contacts are left while the gaps created becomes filled with electrolyte solution which creates a larger solid-liquid interface.

The approach is of using X-ray tomography and X-ray spectro-microscopy together with machine learning to more precisely conduct the statistical analysis. While this combination of techniques appears very powerful the extraction of understanding of the cathode processes stays limited. The authors don't discuss the implications of the described processes or observations, e.g.

Response: We are grateful to reviewer #3 for his/her assessments on the technical aspects of our work. We also appreciate the specific suggestions and concerns raised by reviewer #3. We have studied all the comments and suggestions and have revised our manuscript accordingly and substantially.

(i) In the inital phase of particles becoming detached the battery processes should be enhanced since the particle-electrolyte interface becomes larger. Since the charge carrier concentration is much higher in the solid state compared to the liquid an overall increased activity should be observed. Only in a later stage the contribution of the detaching particle should become deminished.

Response:

We agree with the reviewer that the battery performance enhancement could be the case in the early stage of the particle detachment from the CBD and the reason is in-line with the description by the reviewer.

To illustrate such an effect, we construct a conceptual model to describe the influence of particle detachment on the local conductivity of electron and Li-ion, which is shown in the revised Figure 4e. As discussed in the manuscript, the particle detachment will rearrange the local electrical conductivity and favor the local ionic conductivity as it leads to better contact between the liquid electrolyte and the NMC particle. The competing factors (enhanced ionic conductivity and reduced electrical conductivity) collectively govern the particle's behavior. A particle's actual contribution to the cell level chemistry is dominated by whichever is worse. As a consequence, the performance of the particle could slightly improve in the early stage of the particle detachment due to the improved ionic conductivity. In more severely detached circumstances, the decreased electrical conductivity takes over and results in an overall negative impact.

We add these discussions to the manuscript and changed Figure 4. We also declare here that a more systematic study on such an effect is very valuable. Ideally one would do such evaluation under operating conditions. The herein developed automatic segmentation and quantification methods can well be adapted in the follow-up investigations.

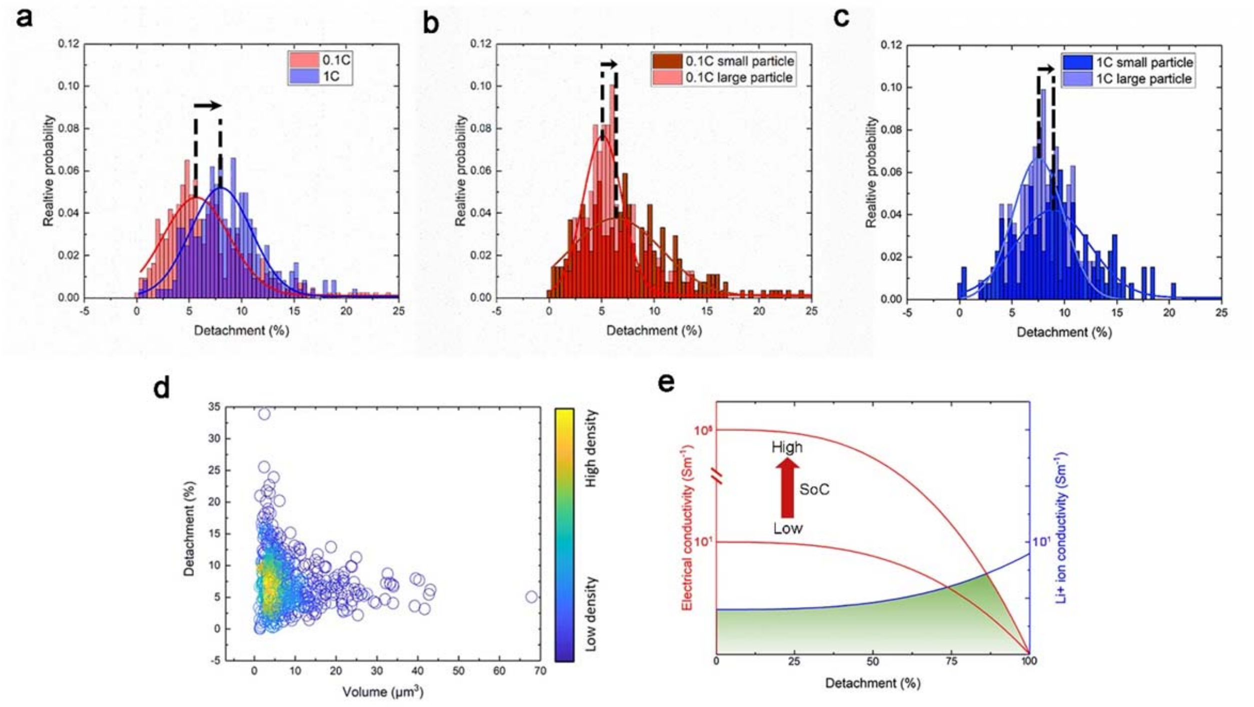

Figure 4. Statistical analysis of the particle detachment. Statistical comparisons of the degree of particle detachment from the CBD matrix are carried out as a function of cycling rate (**a**) and particle size (**b-c**), respectively. The degree of particle detachment is also plotted against the respective particle volume in (**d**) for all the 650 NMC particles studied in this work. **e** Schematic of the changes of the electrical (red) and ionic (blue) conductivity as a function of the particle detachment. The red curve moves up and down depending on the SoC. The particle's actual contribution falls in the green shaded area.

(ii) While the electron density changes with the SoC the authors rightly correlate it with the valence state of Ni. Here it would be useful to give an estimate of the valence change in Fig.5d and/or Fig.5e. In addition, it should be discussed why Mn and Co are not considered to undergo valency changes.

Response: We are grateful for the reviewer's comment. We have benchmarked the color scale of Ni valence map in Figure 5d quantitatively. We conducted this benchmarking by comparing the spectra of the scanned NMC particle to that of the known standards. As shown in Figure R1, the K-edge energy of the Ni XANES spectra positively correlates with the Ni's valence state. Therefore, the Ni's K-edge energy has been broadly utilized as a key descriptor for interpretation of the Ni XANES data.

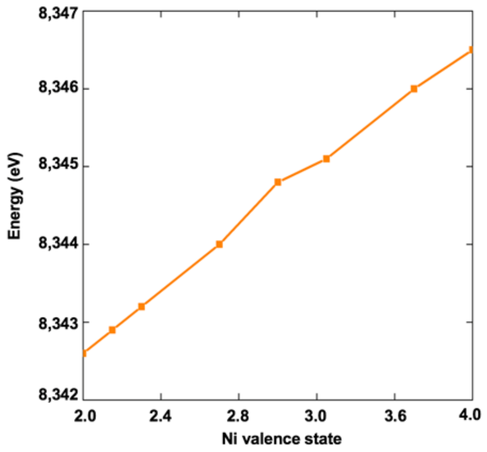

Figure R1. Correlation of the Ni K-edge energy and the Ni's valence state. Data retrieved from Kuppan et al., Nature Communications 8, 14309 (2017).

For NMC811 cathode material, the valence state of Ni cations shows dramatic changes during the charging/discharging process. The Mn^{4+} usually remains electrochemically inactive during charging/discharging and the Co cations only partially contribute to the charge compensation, due to the partial oxidization of Co^{3+} to Co^{4+} and the low Co content in NMC811 material. It has been demonstrated in the literature that the changes in Co XANES are insignificant. [Kim et al., J. Power Sources 2011, 196, 6710−6715; Sun et al., J. Electrochem. Soc. 2011, 159, A1-A5; Tian et al., J. Electrochem. Soc. 2018, 165, A696-A704].

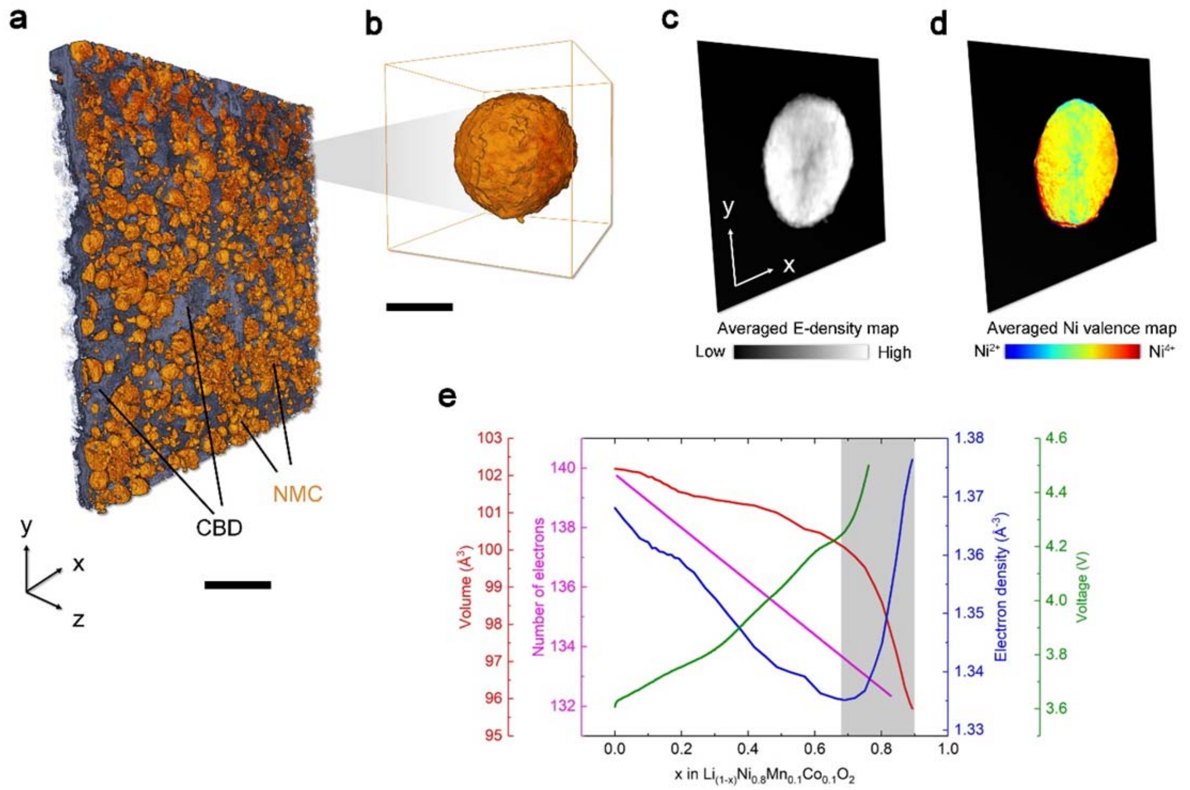

Figure 5. Electron density as a proxy for the SOC. a The 3D rendering of an electrode with a monolayer of NMC 811 particles. **b-d** illustrates our correlative approach for imaging the same NMC particle. **c** is the phase contrast image collected at ID16A, ESRF, Grenoble, France. **d** is the spectroscopic imaging data of the same particle collected at 6-2C, SSRL, Menlo Park, California, USA. **e** shows the changes of lattice unit cell volume, number of electrons per lattice unit cell, voltage (1C) and electron density as a function of the SoC. The scale bars in (**a**) and (**b**) are 25 µm and 5 µm, respectively.

(iii) As stated, 10 charge-discharge cycles were performed at different C-rates. This may considered to be just in the break-in phase, longer cycling would be preferable.

Response: We agree with the reviewer about this. Our statistical analysis of the particle detachment shows that the damage in our sample is still at a relatively low level. While it is desirable to look into the battery electrode that has gone through extensive cycling, we would highlight that our on-going effort to conduct this type of measurements under in-situ conditions, which would eliminate the cell-to-cell variation and facilitate the tracking of the same particles over long term cycling. Our established data analysis tools will play an important role in follow-up studies. Another point we would like to make is that it is very desirable to detect or even to predict the mechanical failure point in the composite electrode in the early state. For the development of this predictive model, it is important to acquire the high-resolution tomography data in both the early cycles and the late cycles.

(iii) All experiments are done ex-situ after disassembling the battery. In addition to saying that in-operando experiments are desirable, it should clearly be discussed where the limitation are with the current procedure and why in-operando experiments would be more revealing.

Response: There are few limitations with the current procedure. First of all, the disassembling process could cause damage to the electrode and, subsequently, affect the result of the statistical analysis. Second, the relaxation of the electrode may lead to charge redistribution in the electrode, making it difficult to evaluate the electrode scale chemical heterogeneity, which could be thermodynamically metastable. Finally, the cell-to-cell discrepancy is a common effect, which could add more complexity to the analysis and interpretation. All these limitations can be tackled by implementing an operando experimental strategy. We are actively pursuing this and our developments in the computational aspects (this paper) will be very useful in the follow-up studies.

The paper is initial work illustrating a novel methodological approach but little discussion of what we learn from it regarding the battery processes. The paper is interesting from an experimental point of view, the scientific content is rather shallow. The paper should not be published in its current form.

Response: We are grateful for the constructive comments. We wish that our substantial revisions of the manuscript have addressed the concerns from the reviewer #3.

A minor point: Why is Fig.1 in gray scale in contrast to all other figures, it is hard to make out the differences.

Response: It is useful to present the imaging data in its most native form in Figure 1. The color-coded rendering of the data relies on accurate segmentation. The superior resolution and image contrast offered by the phase contrast nano-tomography technique lays out the basis for the presented development of ML and modeling. It is our intention to communicate this point by presenting the tomography data without too many cosmetic decorations.

Reviewer #2 (Remarks to the Author):

The authors have addressed my comments by substantially deepening the discussion of their machine learning algorithm. They have also pledged to make the code and data openly available. I think the results presented in the manuscript are novel and key additions to the field. I recommend publication in Nature Communications.
